# Hydrodynamic behaviour of the seawater-freshwater mixing zone induced by cutoff wall installation in sloping coastal aquifers

**DOI:** 10.1038/s41598-026-67038-3

**Published:** 2026-08-20

**Authors:** Asaad M. Armanuos, Kristian Micko, Marcela Bindzárová Gergeľová, Martina Zeleňáková

**Affiliations:** 1 Irrigation and Hydraulics Engineering Department, Faculty of Engineering, Tanta University, Tanta, Egypt; 2 Centre of Digital Technologies in Construction, Faculty of Civil Engineering, Technical University of Kosice, Kosice, 04200 Slovakia; 3 Institute of Geodesy, Cartography and Geographical Information Systems, Faculty of Mining, Ecology, Process Control and Geotechnology, Technical University of Kosice, Kosice, 04200 Slovakia; 4 Institute of Environmental Engineering, Faculty of Civil Engineering, Technical University of Košice, Košice, 04200 Slovakia

**Keywords:** Underground dam, Mixing zone, SWI, Coastal aquifers, Sloped beach, Environmental sciences, Hydrology

## Abstract

Sustainable groundwater management depends on understanding the dynamic behaviour of seawater intrusion (SWI), a slow and persistent phenomenon that might go on for decades in coastal aquifers. A crucial element in regulating groundwater flow and solute transport in coastal aquifers is the freshwater-saltwater mixing zone. The hydrodynamic behaviour of the mixing zone under various coastal aquifer geometries with physical barriers like cutoff walls has not received much attention, despite its significance. The dynamics of the mixing zone are examined in this study by employing numerical simulations, with particular attention paid to the various bottom opening widths (S) beneath cutoff walls and the inclination angles of the sloping beach coastal aquifers (0°, 15°, 30°, and 45°). Regardless of the aquifer slope, findings demonstrate that smaller bottom opening widths result in greater mixing zones and faster inland SWI. The dynamics of the mixing zone were shown to be significantly impacted by the aquifer slope, with steeper slopes (30° and 45°) allowing for more rapid and extensive SWI than flatter slopes (0° and 15°). The largest and fastest-expanding mixing zones, especially over time, were created by a steep slope and a modest bottom opening size.

**Supplementary Information:**

The online version contains supplementary material available at 10.1038/s41598-026-67038-3.

## Introduction

 Due to the salinity variations among freshwater and saltwater, seawater intrusion (SWI) happens naturally^1^. This natural balance is being disrupted in coastal aquifers, however, and is having an adverse effect on the environment and groundwater quality^2^. A variety of approaches have been tried to regulate SWI in coastal aquifers^3^. The efficacy of subterranean barriers has been investigated for avoiding SWI using the FEFLOW model and a physical sandbox model. They discovered that intrusion was avoided by barriers with low permeability^4^. To avoid SWI, Chang et al.^5^ suggested using underground dams having the lowest effective height. Using fresh groundwater discharge, they assessed the approach’s effectiveness and environmental impact. To quantify the equilibrium interface of seawater with subsurface restrictions, Lee et al.^6^ tested hydraulic models. They found that subsurface obstructions like seawalls raised groundwater levels, which improved the pressure gradient and the flow rate and slowed the infiltration of seawater into coastal aquifers. Wu et al.^7^ used SEAWAT to examine how well impervious barriers prevented SWI and found that completely penetrating barriers functioned better than cutoff walls and subsurface dams, and that cutoff walls appeared to be most successful when placed near the coast.

To evaluate the effect of cutoff walls on SWI, Jamshidzadeh and Ghasemzadeh^8^ created a 2D finite difference model. They discovered that cutoff walls significantly reduced the likelihood of SWI. In a cutoff wall test conducted on a laboratory scale, Takahashi et al.^9^ examined the impact of dispersivity on the effectiveness of seawater removal. They found that, in comparison to the experimental results, the leftover saltwater was removed more quickly when low dispersivity was utilised, while greater dispersivity caused the elimination of the residual saltwater to take longer. Zheng et al.^10^ looked at the best location for cutoff walls to stop SWI using a simulation–optimisation technique. They concluded that this approach offers a trustworthy method for figuring out where cutoff walls should be placed. Zheng et al.^11^ investigated the transportation and desalination of seawater after a cutoff wall was installed by using combined numerical simulations as well as laboratory measurements. They discovered that remaining saltwater may be removed more successfully with a deeper cutoff wall. Chang et al.^12^, using a combination of computer calculations and laboratory experimental investigations, studied the impact of cutoff walls on downstream saltwater in coastal aquifers. Dagan and Zeitoun^13^ investigated the effects of aquifer fluctuations on the freshwater-seawater interface using the sharp interface approach.

The knowledge of SWI control mechanisms has greatly increased because of recent experimental and numerical investigations. In their laboratory and computer studies of the impact of aquifer heterogeneity and inclined cutoff walls on seawater wedge dynamics, Emara et al.^14,15^ showed that optimal wall depth and inclination can minimise SWI penetration by as much as 65%. The implications of combined recharge wells and physical barriers in sloping coastal aquifers were simulated using the SEAWAT model^16,17^. They discovered that integrating recharge and barrier methods leads to more successful SWI retreat than using either technique alone. Additionally, the impact on SWI control of groundwater extraction through fractured dams was investigated using SEAWAT code, highlighting the significance of taking well placement, abstraction rates and fracture characteristics into account when designing control measures^18^. Abo-Shaeshaa et al.^19^ investigated the mechanisms and impact of double cutoff walls on the length of the saltwater wedge and the concentrations of nitrates carried downstream from them using a computer model. Because of its vital function in controlling the dynamics of subsurface flow and the exchange of water between groundwater and the ocean, the mixing zone is extremely important. Along the saltwater-freshwater interface, there are variations in the mixing zone width as well as the mixing rate^20^. Mechanical dispersion, molecular diffusion, freshwater discharge, saltwater influx and the density difference between groundwater and saltwater are some of the elements that affect the mixing zone^21^. Regional transverse dispersion has the main cause of the steady state mixing regions^22^.

Recent research has highlighted the growing integration of machine learning techniques with numerical simulation models to assess and mitigate seawater intrusion in coastal aquifers^23–27^,. Abdoulhalik et al.^28^, assessed the capability of subsurface cutoff walls to decrease seawater upconing in coastal aquifers through combined laboratory tests and numerical simulations. Emara et al.^29^, investigated the influence of inclined cutoff walls on controlling seawater intrusion in aquifers. The greatest decrease in seawater intrusion wedge area was achieved with a 45° inclined wall at the highest tested depth ratio. Sun et al.^30^, introduced a new analytical solution for predicting seawater intrusion in coastal aquifers protected by cutoff walls. The adopted model was validated against both numerical simulations and experimental results, viewing strong agreement and reliable performance.

Miao et al.^31^, established a three-dimensional variable-density groundwater model to evaluate and expect seawater intrusion in the coastal region of Longkou City, China. A cutoff wall strategy combined with seawater desalination technology was assessed through an optimization framework aimed at diminishing overall implementation costs. Wu et al.^32^, utilized three-dimensional numerical modeling to observe how subsurface barriers with finite lengths affect seawater intrusion in coastal aquifers. The efficiency of subsurface dams, cutoff walls, and fully penetrating barriers was evaluated under both barrier-only conditions and scenarios that included groundwater abstraction. Zheng et al.^33^, investigated how seawater trapped behind a cutoff wall behaves and gradually disappears after the cutoff wall is implemented for protection of coastal groundwater resources. Armanuos et al.^34^, observed how fractures in subsurface dams affect their capability to control seawater intrusion in coastal aquifers. Using numerical simulations on both benchmark and real-world aquifer systems, the effect of subsurface dam height, location, and fracture characteristics on seawater intrusion control were evaluated.

Luyun et al.^35^, investigated the behavior of residual seawater trapped behind cutoff walls implemented to control seawater intrusion in coastal aquifers. Experimental tests and numerical simulations exposed that the trapped seawater intrusion wedge initially spread and advanced before gradually retreating and being completely flushed from the protected zone. Abdoulhalik et al.^36^, assessed the efficiency of mixed physical barriers that combine a cutoff wall with a subsurface dam to control seawater intrusion in heterogeneous coastal aquifers. Gao et al.^37^, discovered how subsurface dams influence the nitrate contamination and control seawater intrusion in layered coastal aquifers.

The transverse dispersivity determines the width of the saltwater-freshwater interface^38^. A shear effect results from a rise in transverse dispersivity^39^. The mixing zone was shifted seaward at the bottom and landward at the top as a result of this event. Vertical fractures significantly affected the width of the mixing zone but had no effect on the length of the seawater incursion^40^. Transverse dispersion may not have as much of an impact on the mixing zone width as longitudinal dispersion, according to Eeman et al.^41^. As a result, it is unclear how much each longitudinal and transverse dispersion contributes to the freshwater-saltwater mixing zone. Future research on the time-dependent emergence of the mixing zone as well as its effect on convection saline circulation is possibly warranted, as suggested by Narayanan and Eldho^42^. By computing the geometrical average of the two dispersivity values, Abarca and Prabhakar Clement^43^suggested that the simultaneous longitudinal and transverse dispersion significantly influence the mixing zone width. Chang et al.^44^, used numerical simulation and laboratory tests to examine the dynamics of the seawater-freshwater mixing zone and conducted scenarios to compare between the mixing zone variations in cases of with and without subsurface physical barriers. The outcomes demonstrated that the construction of a subsurface barrier will not instantaneously slow down the velocity of seawater intrusion and vary the distribution of salinity for the seawater-freshwater mixing zone. The block effect of cutoff with various bottom opening sizes scenarios became obvious only after the seawater intrusion wedge toe advanced to reach the cutoff wall. Previous studies focused on the impact of physical barriers on the hydrodynamic of mixing zone in vertical beach coastal aquifers and neglected the impact of beach face inclination angle on the mixing zone in the context of cutoff walls. The main objective of this study is to investigate the impact of cutoff wall on the hydrodynamic behaviour of the seawater-freshwater mixing zone in sloping beach faces coastal aquifers. Four various inclination angles have been investigated are 0.0^o^, 15^o^, 30^o^, and 45^o^.The combined impact of the cutoff wall, and the angle of sloping coastal beach on the mixing zone has been investigated by varying the cutoff wall location, bottom opening below cutoff wall, hydraulic conductivity value, despersivity in longitudinal direction, and despersivity in transverse direction. Seven various parameters of the seawater-freshwater mixing zone were examined are the mixing zone (MZ) area, mixing zone bottom width, mixing zone central width, MZ average width, intruded length of 50% isohaline, the length of the 90% isohalines and the submarine groundwater discharge.

## Methodology

**Fig. 1:**
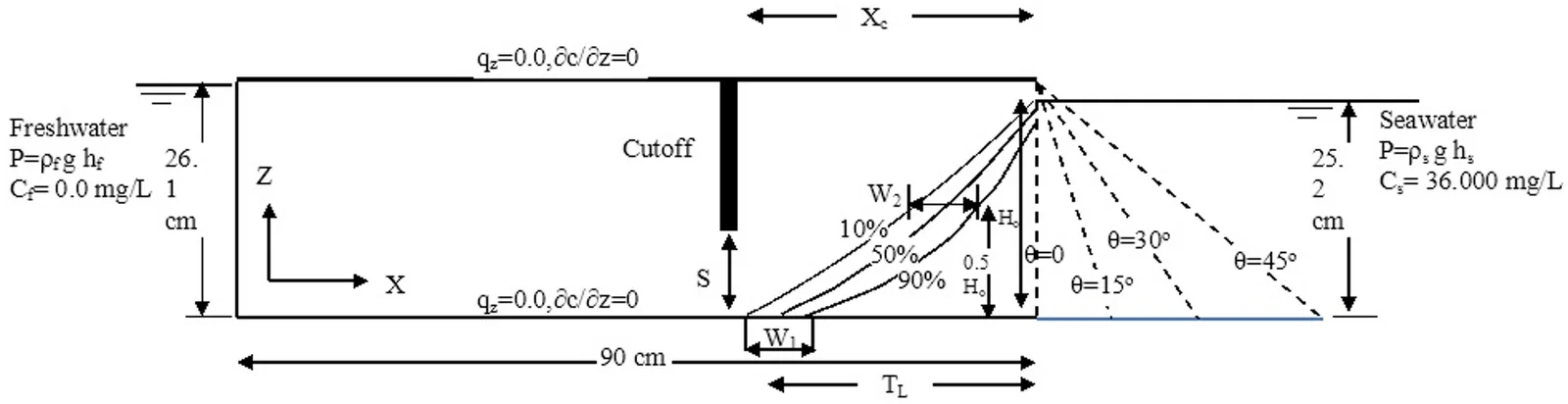
Schematic of the conceptual model. θ represents the inclination angle of the beach, S represents the opening depth under the cutoff, T_L_ represents the saltwater wedge toe length. W_1_ and W_2_ represent the bottom and central mixing zone width, respectively.

 In this study, SEAWAT code^45^ was implemented to simulate SWI in sloping beach coastal aquifers in the context of a cutoff wall as a countermeasure to mitigate SWI. The two-dimensional coastal aquifer of Chang et al.^44^ was studied to investigate the hydrodynamic behaviour of the freshwater-seawater mixing zone in sloping beach coastal aquifers. The dimension of the model domain is 90 cm in the x-direction (Length), 27 cm in the z-direction (height) and 5 cm in the y-direction (width). The dimension of the model domain was made discrete with cell dimensions of 0.5 cm x 0.5 cm. A constant concentration of 36,000 mg/l was assigned to the seawater boundary, and a constant concentration of 0.0 mg/l was assigned to the freshwater boundary. The bottom of the aquifer and the top of the aquifer were set to the case of no-flow boundaries. The freshwater head was set to be 26.1 cm in the freshwater boundary, and the seawater head was assigned to be 25.2 cm in the seawater boundary. The value of hydraulic conductivity of the costal aquifer was set to be 6 × 10^− 3^ m/sec. The porosity of the aquifer domain equals 0.4. The seawater and freshwater densities were assigned to be 1025, and 1000 kg/m^3^, respectively. The viscosity value of the dyed saltwater was 0.001 kg/m.s. The dispersivity value was set to be 0.15 cm in the longitudinal direction and in the transversal direction to be 10% of the longitudinal dispersivity^46^. The value of the molecular diffusion coefficient (D) was set to be equal 1 × 10^− 9^ m^2^/s. The dimension of the model domain cell and dispersivity achieve the criterion of the Péclet number (Pe = 3.3 < 4), was fulfilled, thus guaranteeing numerical stability^47^. In addition, the maximum assigned courant number equals 0.1 to achieve numerical stability. The scenarios of opening size of bottom cutoff wall (S) were 10 cm, 8 cm, 6 cm, 4 cm and 2 cm, respectively. To investigate the impact of the sloping beach coastal aquifer, four varying values of beach inclination (θ) were implemented: 0.0^o^ (vertical boundary), 15^o^, 30^o^ and 45^o^. The scenario of SWI without using a cutoff wall was implemented to compare the effects of cutoff wall on the hydrodynamic behaviour of SWI in sloping unconfined coastal aquifers. The duration of the simulation was set to be equal 180 min, and the time step of the simulation was set to be 1 min. Figure 1 shows the schematic diagram of the studied sloping beach coastal aquifer. Table 1 displays the input hydrological parameters of the sloping beach coastal aquifer.

The domain dimensions were selected based on commonly used configurations in previous coastal aquifer modelling studies and are representative of typical hydrogeological settings^44^. Boundary heads were assigned to reproduce a realistic hydraulic gradient between inland recharge conditions and the coastal sea boundary^44^. The selected hydraulic conductivity value reflects average hydraulic behaviour commonly used in seawater intrusion studies and is consistent with previously published modelling work^44,48^. The selected depths tested of the cutoff wall are 15.2, 17.2, 19.2, 21.2, and 23.2 cm with corresponding depth ratio (H_c_/h_s_) equals 0.60, 0.68, 0.76, 0.84, and 0.90, respectively, consistent with the previously published papers^18,23,27,48,49^, and^34^. The selected cutoff wall depth ratio (H_c_/h_s_) equals 0.60, 0.68, 0.76, 0.84, and 0.90, with corresponding bottom opening size (S) equals 10, 8, 6, 4, and 2cm, respectively, consistent with the previously published papers, and^34^. The locations of the cutoff wall (X_c_) equal 5, 10, 15, and 20 cm, with corresponding well location ratios (X_c_/L_o_) equal 0.11, 0.22, 0.32, and 0.43, respectively, were selected to evaluate different management strategies and compatible with published research^18,23,27,48,49^, and^34^. The tested sloped angles 0.0^o^, 15^o^, 30^o^, and 45^o^ were selected to represent typical geological conditions observed in coastal sedimentary aquifers and consistent with published research^50–52^.

### Evaluation indices

A thorough description of the location and extent of the mixing zone is necessary for the appropriate management of coastal groundwater resources. Seven varying parameters have been implemented to evaluate the extent of the mixing zone in a sloping beach coastal aquifer under different scenarios of beach inclination and bottom opening of the cutoff wall. The symbol T_L_ represents the length of the SWI wedge toe, defined as the horizontal distance between the saltwater boundary of the sloping beach coastal aquifer and the 50% isohaline (50% of the seawater concentration in the seawater boundary), which was implemented to assess the extent of SWI. The abbreviation SGD represents the submarine groundwater discharge in the coastal aquifer. The symbol L_90%_ represents the length of the 90% normalised isohalines of the concentration of the seawater boundary. Using the 10% and 90% normalised isohalines, the freshwater-seawater mixing zone can be evaluated^43,53^. The area of the mixing zone (A_M_) is the area in between the 10% and 90% isohalines of the concentration of seawater boundary. The width of the freshwater-seawater mixing zone was represented by W_1_ and W_2_. The width of the mixing zone (W_1_) is the horizontal distance between the distinct isohalines at the bottom of the sloping beach coastal aquifer, and the width of the mixing zone (W_2_) is the horizontal distance between the distinct isohalines at the midpoint of saturated depth of the sloping beach coastal aquifer^39^. The average freshwater-seawater mixing zone width (MZW) as a metric to evaluate the extent of the entire mixing zone equals the A_M_ divided by L_90_% (The length of the 90% isohaline)^54^. The Zone Budget utility in SEAWAT was used to automatically compute the total discharge across selected boundary zones Table 1.

**Table 1:** Defined parameters.

Parameter	Definition
C_s_	Concentration of saltwater
C_f_	Concentration of freshwater
h_s_	Head of seawater
h_f_	Head of freshwater
ρ_s_	Density of seawater
ρ_f_	Density of freshwater
D	Molecular Diffusion coefficient
H_c_	Depth of cutoff wall
H_c_/h_s_	Depth of cutoff wall ratio
S	Opening size below the cutoff wall
X_c_	Cutoff wall distance measured from seawater boundary
X_c_/L_o_	Cutoff wall location ratio
θ	Inclination angle of sloping beach coastal aquifer
K	Hydraulic conductivity of the aquifer
H_a_	Saturated aquifer depth at the sea boundary
L_o_	Initial intruded length of seawater intrusion wedge
L_T_	Intruded length of 50% isohaline
L_90%_	The length of the 90% normalized isohalines of SWI
W_1_	Width of the mixing zone at the bottom of the aquifer
W_2_	Width of the mixing zone at 50% of the saturated aquifer depth
MZW	Average mixing zone width
SGD	Subsurface groundwater discharge
A_M_	Area of the mixing zone
α_L_	Dispersivity in the longitudinal direction
α_T_	Dispersivity of the transverse direction

## Mesh discretization and model verification

To evaluate the sensitivity of the SEAWAT model to grid discretization, simulations were performed using several mesh sizes of 0.5 × 0.5, 1.0 × 1.0, 2.0 × 2.0, 3.0 × 3.0, and 4.0 × 4.0 cm. The model performance was assessed by comparing the simulated seawater intrusion wedge lengths with the corresponding experimental measurements reported by Chang et al.^44^ for the 18,000 mg/L and 3,600 mg/L concentration contour lines. The absolute percentage error between the experimental and simulated seawater intrusion wedge lengths was calculated to quantify model accuracy under different grid sizes. Fig. A1 demonstrates the variation in the calculated error for both the 18,000 mg/L and 3,600 mg/L concentration contours as a function of grid size. The results demonstrate that finer grid discretization leads to improved agreement between numerical and experimental observations, thereby enhancing the accuracy of the SEAWAT simulations. For the 18,000 mg/L contour line, the absolute percentage errors between the experimental and simulated seawater intrusion wedge lengths were 0.7%, 5.4%, 11.2%, 22.9%, and 34.5% for grid sizes of 0.5, 1, 2, 3, and 4 cm, respectively. These values correspond to model accuracies of 99.3%, 94.6%, 88.8%, 77.1%, and 65.5%. Similarly, for the 3,600 mg/L contour line, the calculated absolute percentage errors were 0.4%, 6.4%, 15.5%, 21.2%, and 26.9% for the same sequence of grid sizes. The corresponding model accuracies were 99.6%, 93.6%, 84.5%, 78.8%, and 73.1%, respectively (Fig. A1 in Appendix). These findings confirm that decreasing the grid size significantly improves the predictive capability of the SEAWAT model.

Figure A2 presents a comparison between the experimentally observed seawater intrusion wedge reported by Chang et al.^44^and the corresponding numerical results obtained from the SEAWAT model developed in the present study. This comparison was conducted to validate the model and assess its capability to reproduce the evolution of seawater intrusion over time. The verification process focused on the 18,000 mg/L concentration contour, with evaluations performed at simulation times of 5, 25, and 180 min. At 5 min, the simulated toe length of the saltwater wedge along the base of the coastal aquifer reached 11.51 cm, whereas the experimental measurement was 10.96 cm, resulting in a difference of 0.55 cm. After 25 min of simulation, the intrusion length predicted by the numerical model increased to 24.2 cm compared with an experimentally observed value of 24.96 cm, yielding an absolute error of 0.76 cm. At the final simulation time of 180 min, the numerical model estimated a seawater intrusion toe length of 46.69 cm, while the corresponding experimental value was 46.39 cm. The RMSE between the simulated and experimentally measured wedge lengths from (Chang et al.^44^, equal 0.629, 0.096, and 0.048 cm for the selected times 5, 25, and 180 min, respectively. The discrepancy between the two results was limited to 0.30 cm. Overall, the close agreement between the simulated and measured intrusion lengths at all investigated time intervals demonstrates the reliability of the SEAWAT model in representing the transient behaviour of seawater intrusion within the coastal aquifer system.

## Results and Discussion

### Hydrodynamic behaviour of the seawater-freshwater mixing zone in sloping beach coastal aquifers

The saltwater intrusion (SWI) in coastal aquifers is strongly influenced by both beach inclination and the bottom opening of a cutoff wall. Across all investigated conditions, the saltwater wedge initially develops slowly, while the freshwater-seawater mixing zone remains relatively narrow. As time progresses, seawater moves inland, interactions with the cutoff wall become stronger, and the mixing zone expands until a relatively stable condition is reached after approximately 180 min. For the aquifer with a horizontal beach (0° inclination) (Fig. A3), the absence of a cutoff wall allows the saltwater wedge to advance inland with limited obstruction. When a cutoff wall is introduced, its influence depends strongly on the size of the bottom opening. A large opening, such as 10 cm, produces only a minor effect during the early stages. However, reducing the opening progressively restricts seawater movement and modifies the groundwater flow field. Openings of 8, 6, 4, and 2 cm increasingly delay the saltwater wedge and cause greater enlargement of the mixing zone. The smallest opening produces the strongest flow disturbance because freshwater and seawater are forced to interact within a more restricted region, increasing dispersion and widening the interface. Increasing the beach inclination to 15° intensifies these effects (Fig. A4). The slope redirects groundwater flow and produces a more pronounced upward movement of the mixing zone. Consequently, the influence of smaller cutoff-wall openings becomes more evident, particularly after 30 and 180 min. With 6 cm, 4 cm, and 2 cm openings, the saltwater wedge is increasingly delayed, while the mixing zone becomes wider and more vertically displaced. The inclined geometry also creates a less uniform distribution of the mixing zone compared with the horizontal configuration.

**Fig. 2:**
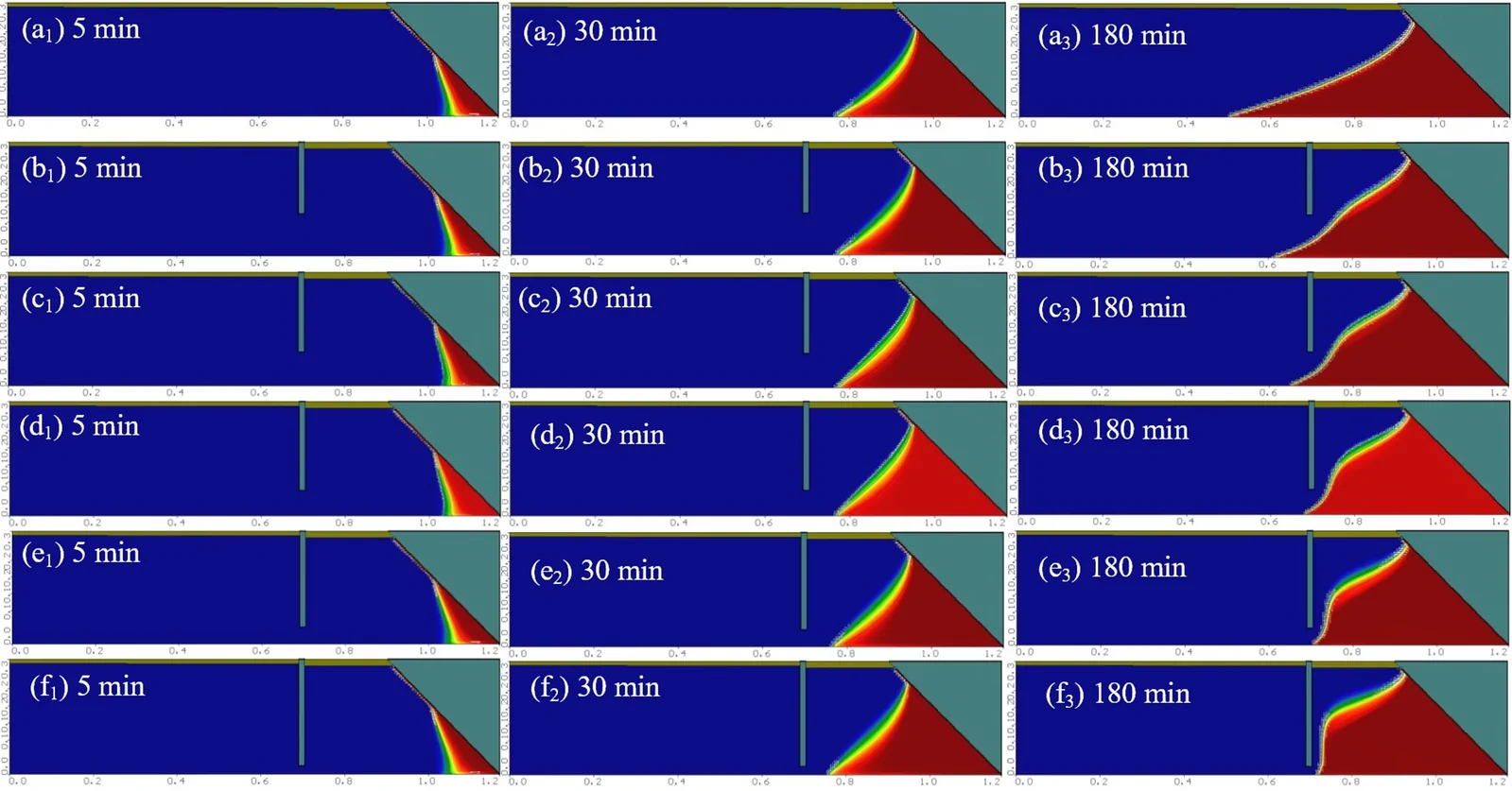
Steady state seawater intrusion for a sloped beach coastal aquifer with an inclination angle of 45^o^ for (a) without a cutoff wall, and for different values of space below the cutoff wall S: (b) 10 cm, (b) 8 cm, (b) 6 cm, (e) 4 cm and (f) 2 cm; the black lines indicate the 10, 50 and 90% isohalines of the mixing zone.

At 30° inclination (Fig. A5), the upward movement of the freshwater–seawater interface becomes even more prominent. The cutoff wall increasingly obstructs the advancing saltwater wedge as the opening decreases. The 2 cm opening produces the strongest restriction, resulting in substantial lateral and vertical expansion of the mixing region by the final simulation time. Thus, both the beach slope and the reduced opening work together to intensify flow distortion and saltwater dispersion. The 45° configuration produces the most pronounced response (Fig. A6). Even with larger openings, the sloping geometry causes stronger upward displacement of the interface. Smaller openings substantially delay intrusion and generate a considerably broader, more asymmetric mixing zone. The 2 cm opening produces the greatest disruption, with pronounced vertical and lateral expansion after 180 min. Overall, the simulations indicate that cutoff walls can effectively modify SWI, but their influence is governed primarily by opening size and beach inclination. As the bottom opening becomes narrower, seawater migration is increasingly restricted, freshwater flow is redirected, and the mixing zone expands. Increasing beach inclination further amplifies these effects, with the 45° slope producing the strongest vertical displacement and asymmetry. Therefore, combining a cutoff wall with an appropriately designed bottom opening can significantly alter the extent and development of saltwater intrusion in sloping coastal aquifers Fig. 2.

The results of Figs. A7, A8, A9, and A10 demonstrates that both coastal aquifer slope and cutoff-wall bottom opening size strongly control seawater intrusion (SWI) and mixing-zone development. Increasing the beach slope from 0° to 45° enhances hydraulic gradients, accelerating seawater movement inland and increasing lateral and vertical dispersion. Conversely, reducing the bottom opening from 10 cm to 2 cm generally produces a wider and more rapidly developing mixing zone due to greater freshwater–saltwater interaction. Among the tested configurations, the 45° slope with a 2 cm opening produces the most extensive mixing zone and fastest inland seawater migration, highlighting the combined influence of slope and opening size on SWI dynamics (Figs. A7, A8, A9, and A10).

## Hydrodynamic behaviour of subsurface groundwater discharge in sloping beach coastal aquifers

**Fig. 3:**
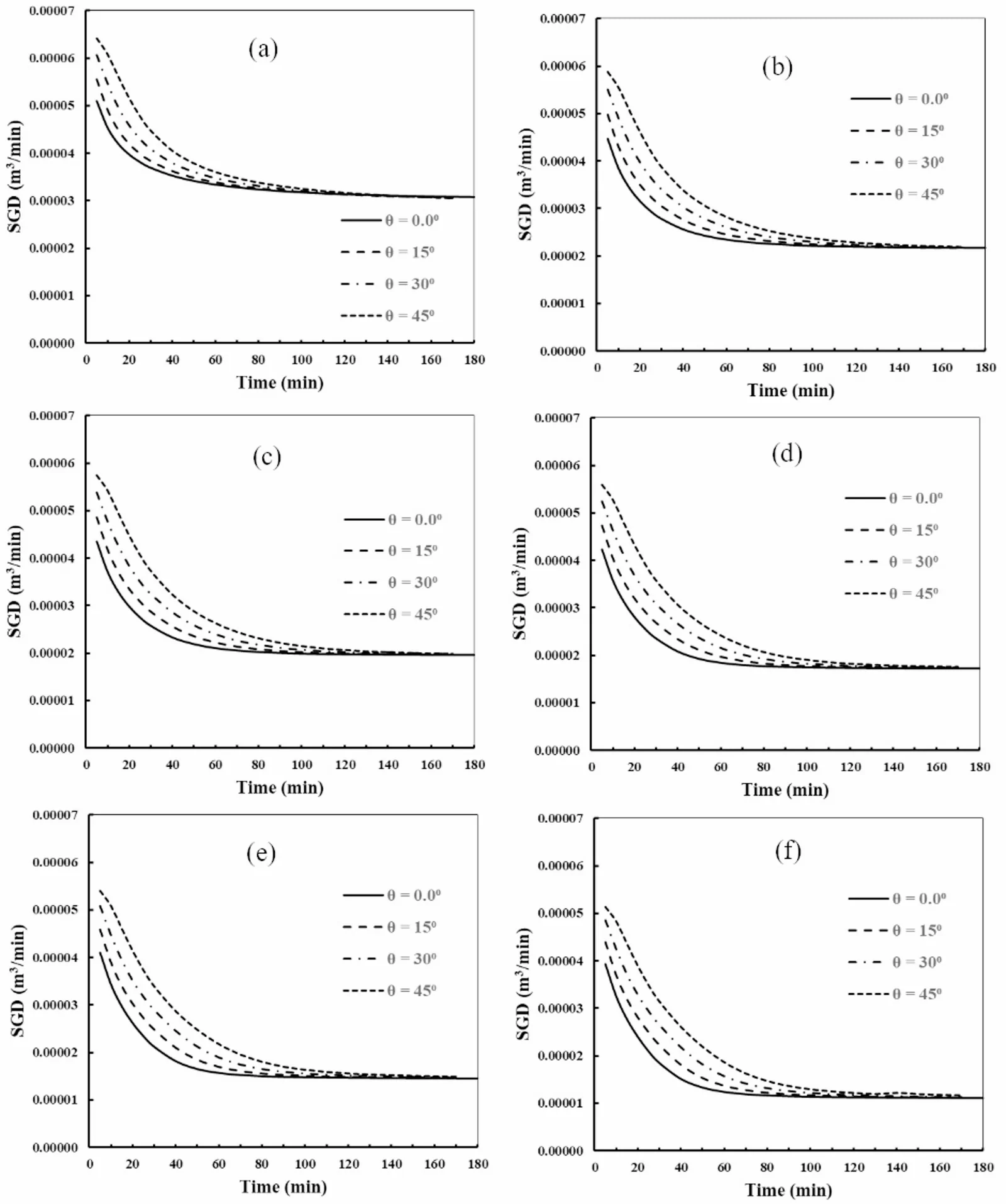
Hydrodynamic behaviour of subsurface groundwater discharge (SGD) for different values of sloping beach coastal aquifers θ = 0.0, 15, 30, and 45 for different cases of cutoff: (a) No cutoff, (b) S = 10 cm, (c) S = 8 cm, (d) S = 6 cm, (e) S = 4 cm, and (f) S = 2 cm.

The hydrodynamic behaviour of subsurface groundwater discharge (SGD) in coastal aquifers with varied inclination angles (θ) (θ = 0°, 15°, 30°, and 45°) is shown in Fig. 3 for various bottom opening sizes (S) (S = 2 cm, 4 cm, 6 cm, 8 cm, and 10 cm) of the cutoff wall. In all slope circumstances, there is a strong initial reduction in the SGD, which then gradually decreases and approaches a steady state after 120 min. By increasing with slope angle, the initial SGD (at time = 0 min) rises from almost 0.00004 m³/min at θ = 0° to nearly 0.000065 m³/min at θ = 45°, signifying a 62.5% increase between the lowest and highest slopes. The SGD decreases more quickly in the early hours on steeper slopes, with the rate of loss being most noticeable during the first 60 min. There is less than 10% difference between the various slope scenarios by the end of the simulation period (180 min), when the SGD values on all slopes converge to a small range of about 0.00003 m^3^/min. SWI into the aquifer is progressively being lessened, as seen by the SGD values’ slow decline and final steady state. This behaviour is consistent with the findings of Chang et al.^44^, which show that when the saltwater wedge gets closer to the barrier, the impact of subsurface physical barriers, such as cutoff walls, increases over time. At first, the SGD rapidly decreases for all values of S and θ. This shows how the physical barrier is immediately limiting the intrusion of seawater. The SGD stabilises, and the rate of the decline reduces with time, indicating a balance in the interplay between freshwater and saltwater at the mixing zone. This time-dependent effect demonstrates how a cutoff wall progressively modifies groundwater discharge as the mixing zone changes rather than instantly altering the velocity of seawater invasion. The rate at which the SGD decreases is strongly influenced by the coastal aquifer’s inclination angle. Since the flow path is more direct and the cutoff wall can have a greater immediate impact on the groundwater flow, the SGD reduces comparatively more quickly for θ = 0° (vertical beach aquifer) than for the other angles. The SGD falls more slowly at greater inclinations (15°, 30° and 45°). The cutoff wall’s initial efficacy in preventing SWI is diminished due to the more complicated flow dynamics caused by the increasing slope. When the aquifer is sloped, the physical barriers have a delayed effect on the saltwater wedge, causing the overall hydraulic gradients to vary as the angle increases and slowing down the stabilisation of the SGD. Smaller Bottom Openings (S = 2 cm, 4 cm): These situations exhibit a more pronounced drop in the SGD, particularly in the early phases, since the cutoff wall can block SWI more rapidly due to the more effective flow restriction provided by smaller openings. Larger Bottom Openings (S = 6 cm, 8 cm, and 10 cm): The SGD reduces more slowly as the opening size grows. More seawater can enter via larger apertures before the barrier’s effects are apparent. The larger openings cause a more progressive decrease in the SGD over time, indicating that the size of the bottom opening is a critical factor in determining how well the cutoff wall controls seawater invasion.

The evolution of the seawater-freshwater interaction is influenced by the coastal aquifer’s inclination angle (θ); steeper angles cause the hydraulic gradients to change, which slows down the response in the SGD. The findings shown in Fig. 3 show that the inclination angle (θ) of the coastal aquifer and the bottom opening size (S) of the cutoff wall have a significant impact on the hydrodynamic behaviour of the SGD. The SGD decreases more quickly in aquifers with smaller bottom openings and a vertical beach aquifer, but more slowly in aquifers with bigger openings and steeper slopes. These results demonstrate how SWI processes are time-dependent and how well subsurface physical barriers work to control the dynamics of coastal aquifers. Figure 3 displays the temporal variation of SGD under various inclination beach faces angles (θ = 0°, 15°, 30°, and 45°). In all tested cases, SGD decreases rapidly during the initial stage and gradually approaches a steady state condition with increasing time. At the beginning of the numerical simulation, the SGD varies from approximately 4.5 × 10^− 4^ to 6.5 × 10^− 4^ m^3^/min, depending on the inclination angle of coastal aquifer beach. Increasing the inclination angle from 0° to 45° results in an increase in the initial SGD of about 20–40%, with the largest differences observed during the first 20–40 min. For example, in Fig. 3(a), where no cutoff wall, the SGD at θ = 45° is approximately 6.3 × 10^− 4^ m³/min, compared with about 4.8 × 10^− 4^ m³/min at θ = 0°, corresponding to an increase of nearly 31%. As the time progresses, the discrepancy between the curves gradually decreases. After approximately 100–120 min, all tested scenarios converge toward similar steady-state condition values, varying from about 1.1 × 10^− 4^ to 3.1 × 10^− 4^ m³/min depending on the case, and the relative difference between inclination beach face angles becomes less than 5%. These results show that the impact of inclination angle is most pronounced during the early stage of SGD evolution, whereas its effect becomes negligible as the seawater intrusion approaches equilibrium.

### Hydrodynamic behaviour of SWI wedge length (50%) in sloping beach coastal aquifers

**Fig. 4:**
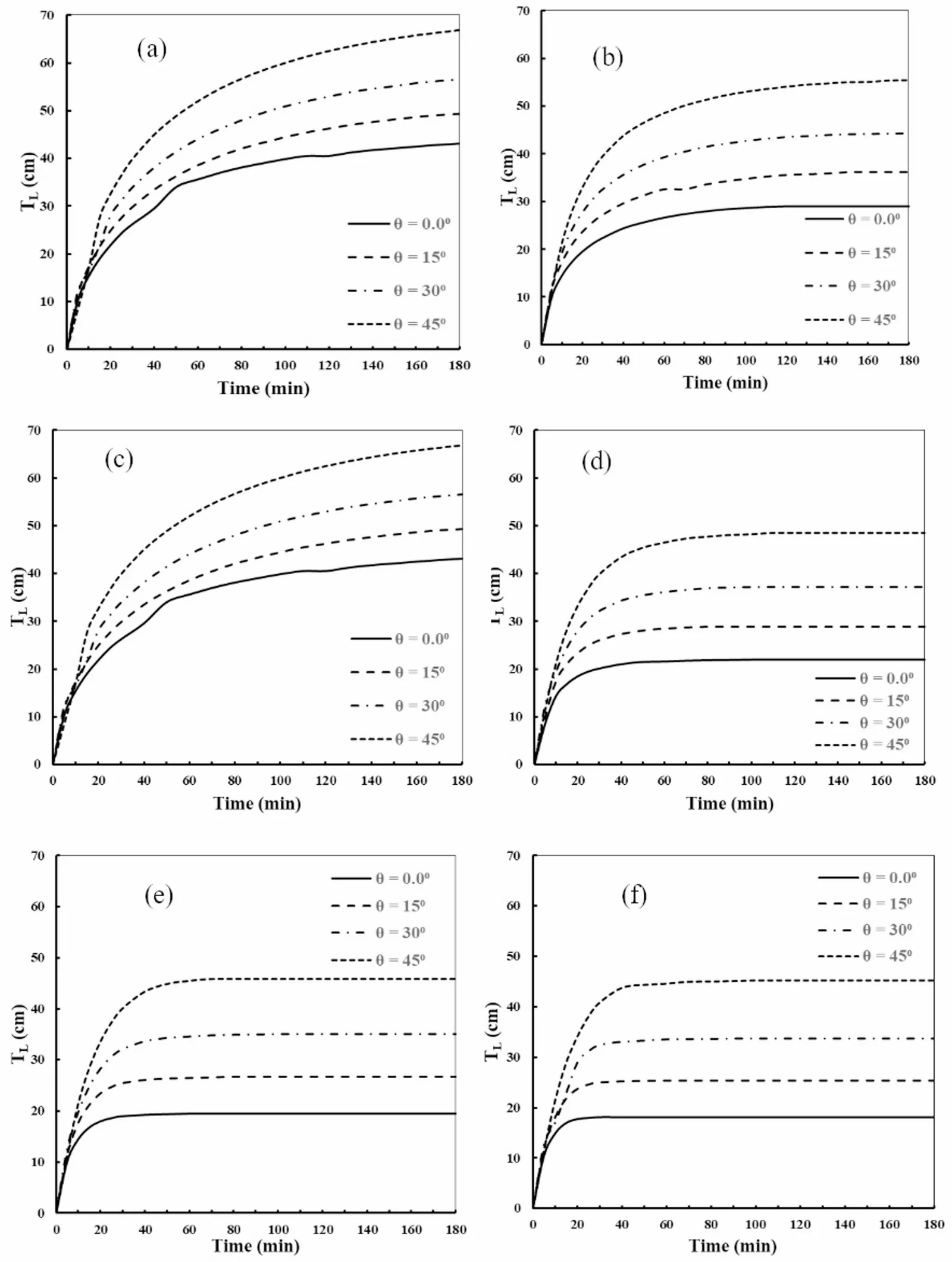
Hydrodynamic behaviour of the SWI wedge length (50%) for different values of sloping beach coastal aquifers θ = 0.0, 15, 30 and 45 for different cases of cutoff: (a) No cutoff, (b) S = 10 cm, (c) S = 8 cm, (d) 6 cm, (e) 4 cm and (f) 2 cm.

The hydrodynamic behaviour of the SWI wedge length (T_L_) in sloping beach coastal aquifers is depicted in Fig. 4 for a range of bottom opening sizes (S) of the cutoff wall. The figure comprises six subfigures, (a) through (f) that, under various inclination angle conditions (θ = 0°, 15°, 30° and 45°) and cutoff wall bottom opening sizes (S = 2 cm, 4 cm, 6 cm, 8 cm and 10 cm), illustrate the variation of the SWI wedge length (T_L_) over time (in minutes). In every case, T_L_ rises quickly in the beginning and then more gradually towards a quasi-steady state over time. All slope situations show a similar pattern at the beginning, although T_L_ advances much more quickly on steeper slopes (θ = 45°). By 60 min, T_L_ had increased by almost 63% compared to the θ = 0° slope condition, reaching about 38 cm for θ = 0° and 62 cm for θ = 45°. This suggests that the extent of SWI and beach slope angle is positively correlated, especially in the early transitory phases. Maximum penetration distances were found to be roughly 42 cm, 50 cm, 56 cm and 66 cm for θ = 0°, 15°, 30° and 45°, respectively, as T_L_ stabilises by the end of the observation time (180 min). The process of SWI into the aquifer is characterised by an increase in the SWI wedge length (T_L_) over time. When SWI into the freshwater aquifer occurs, the wedge length first grows quickly. The wedge length becomes closer to a steady state as time goes on, and the rate of expansion slows. The SWI moves more slowly as it gets closer to equilibrium after initially moving swiftly, especially in the presence of a physical barrier like a cutoff wall. The influence of the cutoff wall becomes increasingly noticeable with time, as evidenced by the time-dependent increase in T_L_.

The cutoff wall restricts the seawater’s progress, and as the seawater wedge is progressively obstructed by the barrier, this restriction becomes more apparent later in the simulation. Long-term wedge length stabilisation supports the finding in case of vertical beach face that cutoff walls do not instantly prevent SWI but rather progressively lessen its magnitude as the barrier’s influence grows over time. Over time, the SWI wedge length is strongly influenced by the inclination angle (θ). The seawater wedge length increases the fastest in the θ = 0° (vertical beach aquifer) scenario because the flow is more consistent and SWI is initially less obstructed. In the early stages, the seawater wedge rises more slowly as the inclination angle increases (15°, 30°, and 45°), especially in the steeper aquifers (θ = 45°). Hydraulic gradients steepen with increasing inclination angle, slowing the invasion rate and resulting in a more gradual increase in the seawater wedge length. The delayed reaction in SWI caused by the steeper inclination angles (15°, 30°, and 45°) indicates that the effect of the cutoff wall becomes more noticeable over time.

This is in line with Chang et al.^44^, who observed that as the saltwater wedge approaches the barrier and the barrier’s influence intensifies over time, the impact of the physical barrier on SWI gets more noticeable. The length of the SWI wedge is mostly dependent on the cutoff wall’s bottom opening size (S). The rate at which T_L_ grows slows down as the size of the bottom hole increases. Because the cutoff wall can more successfully prevent SWI in the early phases of the simulation with smaller bottom apertures (S = 2 cm, 4 cm), the seawater wedge length increases more quickly at first. The seawater wedge increases more slowly for bigger bottom openings (S = 6 cm, 8 cm, and 10 cm), suggesting that more seawater can intrude before the barrier significantly slows down the process. The slow rise in T_L_ in these situations suggests that the effect of the cutoff wall on SWI is not immediately apparent in larger openings. The results of Fig. 4 demonstrated that SWI processes are time-dependent and that the impact of subsurface physical barriers, or cutoff walls, on SWI increases with the saltwater wedge’s proximity to the barrier. The paper stressed that the size of the cutoff wall’s bottom opening has a major impact on both the rate of SWI and how well the barrier restricts infiltration over time. This is seen in the figure, as larger openings permit more SWI before stabilising, whereas smaller openings cause the seawater wedge to stabilise more quickly. Because steeper aquifers displayed a more gradual growth of SWI, the inclination angle also influenced SWI dynamics. This is further supported by the figure, which shows slower increases in wedge length at steeper slopes.

Figure 4 illustrates that seawater intrusion wedge length (T_L_) increases rapidly during the early stage of the simulation and gradually approaches a stable value with time for all tested cases of inclination angles. Higher inclination angles consistently produce larger (T_L_) values. In Fig. 4(a), the final (T_L_) increases from approximately 43 cm at θ = 0° to about 67 cm at θ = 45°, corresponding to an increase of nearly 56%. Similar trends are observed in the other cases, where the increase in (T_L_) between θ = 0° and θ = 45° ranges from approximately 55% to 150%. The differences among inclination angles become more pronounced with time and remain evident at steady state, indicating that inclination angle has a substantial and persistent impact on the development of (T_L_).

### **Hydrodynamic behaviour of the bottom width of the mixing zone (W**_**1**_**) in sloping beach coastal aquifers**

**Fig. 5:**
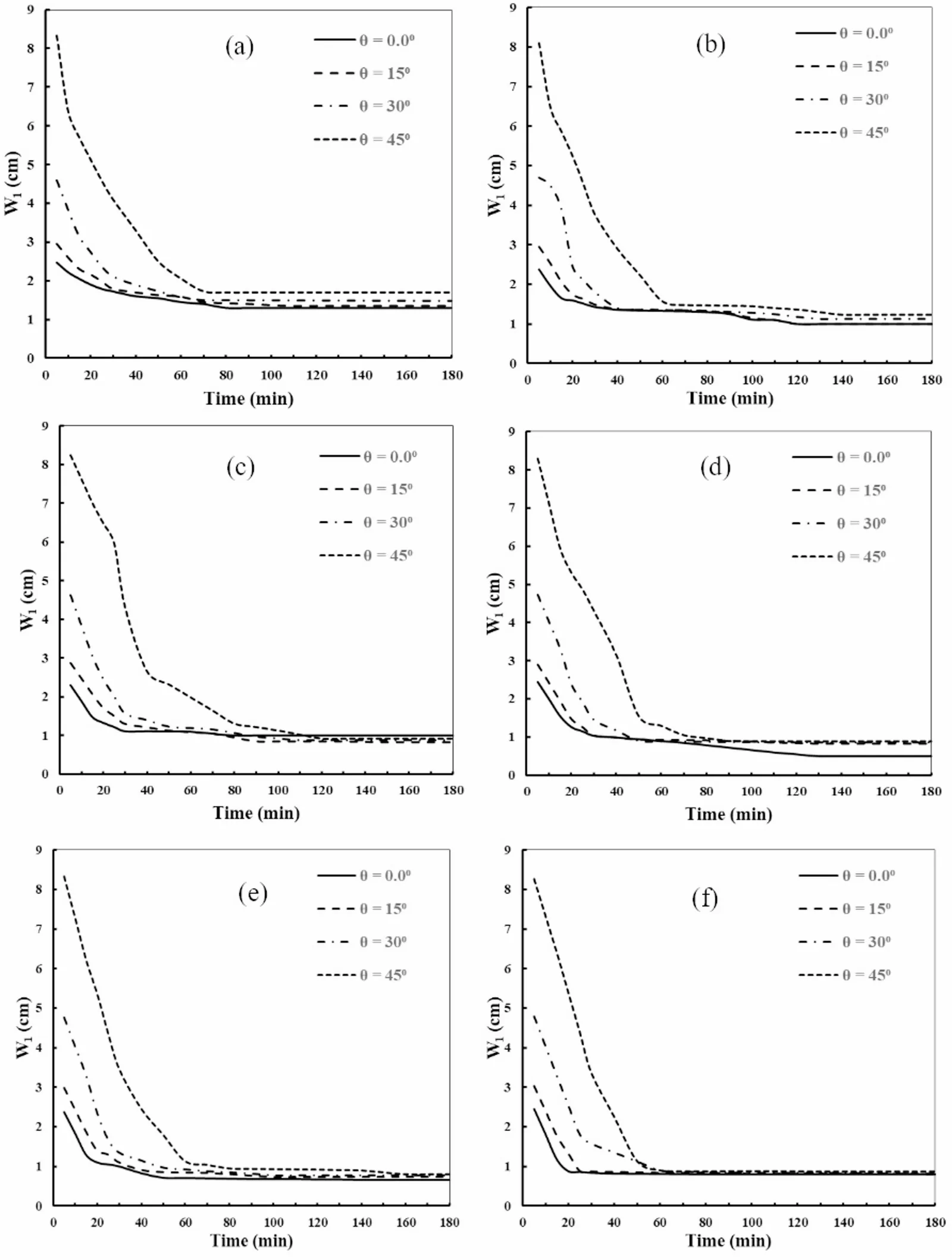
Hydrodynamic behaviour of the bottom width of the mixing zone (W_1_) for different values of sloping beach coastal aquifers θ = 0.0, 15, 30 and 45 for different cases of cutoff: (a) No cutoff, (b) S = 10 cm, (c) S = 8 cm, (d) 6 cm, (e) 4 cm and (f) 2 cm.

The hydrodynamic behaviour of the mixing zone’s bottom width (W_1_) over time for varying cutoff wall bottom opening sizes (S) in sloping beach coastal aquifers is depicted in Fig. 5. Six subfigures, (a) through (f), in the figure illustrate how the mixing zone width (W_1_) changes over time for various inclination degrees (θ = 0°, 15°, 30° and 45°) and cutoff wall bottom opening sizes (S = 2 cm, 4 cm, 6 cm, 8 cm and 10 cm). As seawater intrudes into the freshwater aquifer and the mixing zone forms, the bottom width of the zone (W_1_) in all subfigures first rapidly diminishes with time. The rate at which the width decreases then slows with time, and the mixing zone stabilises. The creation of equilibrium between the freshwater and saltwater interactions is reflected in this slow stabilisation in W_1_. For all slope situations, there is initially a significant drop in W_1_, with steeper slopes showing much larger initial discharge widths. W_1_ shows a sharp rise from the lowest to the highest slope angle at time zero, ranging from almost 2 cm for the horizontal slope (θ = 0°) to nearly 8.5 cm for the steepest slope (θ = 45^o^). Within the first 60 min, the discharge breadth rapidly decreases before progressively stabilising. W_1_ approaches near-equilibrium values for all slopes by about 80 min. With less than 20% relative variance among slope conditions at equilibrium (120–180 min), W_1_ converges to roughly 1.4–1.8 cm, suggesting that the influence of slope angle diminishes with longer time periods.

The extension of the mixing zone is significantly influenced by the coastal aquifer’s inclination angle (θ). In the first stage, the mixing zone width drops the fastest for θ = 0° (vertical boundary aquifer), indicating that SWI is more effective, and the horizontal flow is less obstructed. The mixing zone width reduces more slowly as the inclination angle rises (15°, 30° and 45°). The mixing zone declines more gradually in steeper aquifers (higher θ values) because they produce more intricate hydraulic gradients and flow patterns. Because seawater penetration becomes less direct and more reliant on the changed flow dynamics in the aquifer, the physical barrier’s impact is more delayed in these situations. This finding demonstrated that steeper aquifers have more intricate flow patterns, which cause the width of the mixing zone to change more slowly as the SWI process progresses. Lower Bottom Openings (S = 2 cm, 4 cm): At first, the mixing zone width (W_1_) drops more slowly. The cutoff wall’s narrower bottom openings serve as a more efficient barrier to SWI, halting the mixing zone’s rapid growth. According to Chang et al.^44^, smaller bottom apertures have a more immediate and apparent effect on decreasing SWI and narrowing the mixing zone breadth, which is consistent with this behaviour. The mixing zone width decreases more gradually in situations with larger bottom openings (S = 6 cm, 8 cm and 10 cm). The effect of the physical barrier is first less noticeable since larger bottom apertures make it easier for seawater to seep into the aquifer. Because the larger opening for more seawater flow before the cutoff wall starts to affect the mixing zone’s behaviour, the mixing zone widens more slowly in the early phases. Figure 5 demonstrates the temporal evolution of the bottom width of the mixing zone (W_1_) under various inclination angles. In all cases, (W_1_) decreases rapidly during the initial stage of the simulation and gradually approaches a nearly constant value. The largest reductions occur at θ = 45°, where (W_1_) decreases from approximately 8.3–8.5 cm to 0.8–1.7 cm, corresponding to a reduction of about 80–90%. For θ = 0°, the initial values of (W_1_) are considerably lower (about 2.5–3.0 cm) and decline to approximately 0.7–1.4 cm. Most of the decrease occurs within the first 40–80 min, after which the curves stabilize and the differences among inclination angles become relatively small. These results indicate that increasing the inclination angle primarily affects the decline of (W_1_), while its influence diminishes as the seawater intrusion approaches steady state condition. The pace at which the mixing zone widens is strongly influenced by the cutoff wall’s bottom opening size (S). While larger openings permit more gradual changes, smaller openings cause the mixing zone to stabilise more quickly. The flow dynamics and, in turn, the rate of mixing zone widening are influenced by the inclination angle (θ). Steeper aquifers respond more slowly because the cutoff wall’s immediate effectiveness is hampered by more intricate flow patterns.

### **Hydrodynamic behaviour of the mixing zone width (W**_**2**_**) at average saturated depth in sloping beach coastal aquifers**

**Fig. 6:**
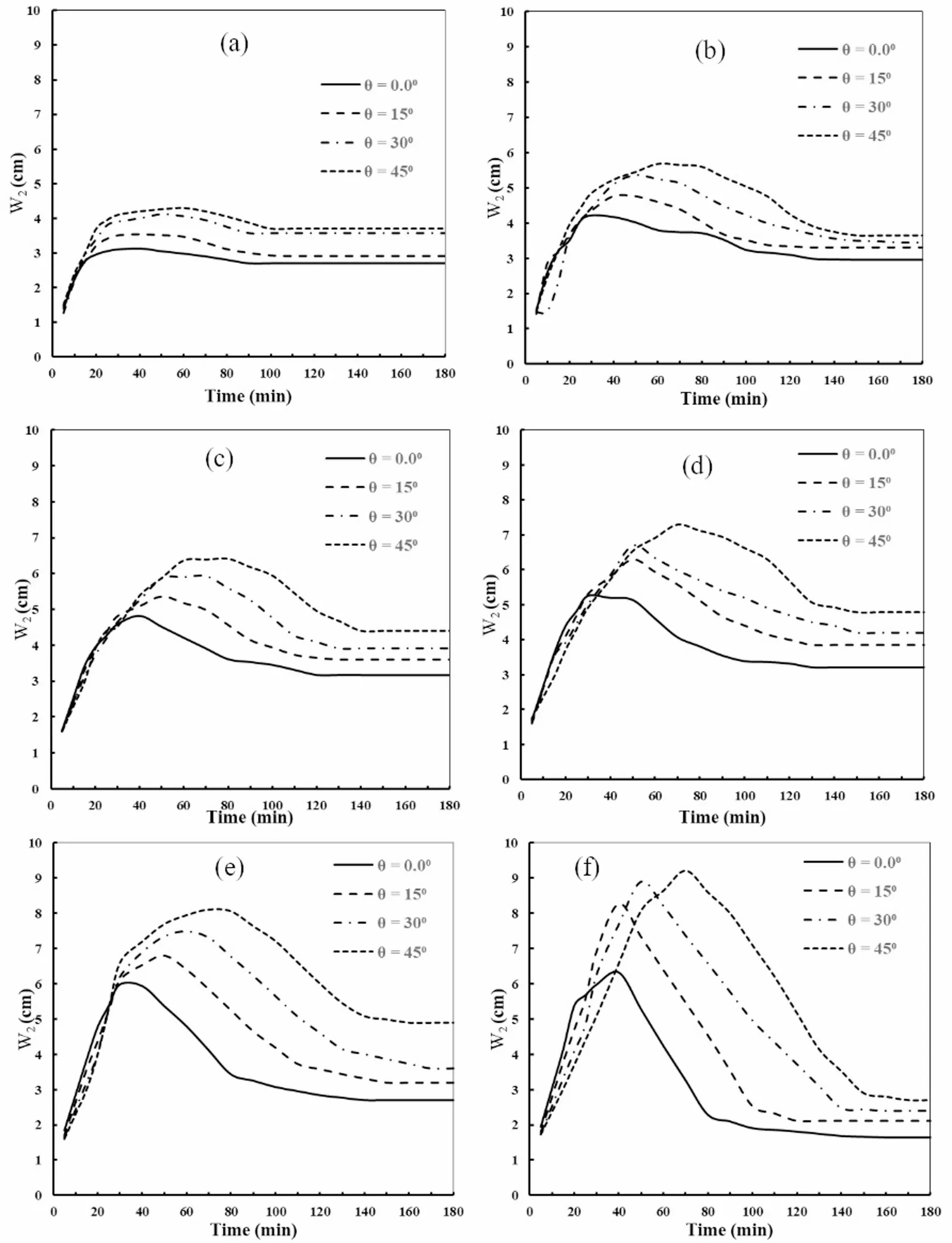
Hydrodynamic behaviour of the width of the mixing zone (W_2_) at average saturated depth for different values of sloping beach coastal aquifers θ = 0.0, 15, 30 and 45 for different cases of cutoff: (a) No cutoff, (b) S = 10 cm, (c) S = 8 cm, (d) 6 cm, (e) 4 cm and (f) 2 cm.

The hydrodynamic behaviour of the mixing zone width (W_2_) at the average saturated depth over time is shown in Fig. 6 for a range of cutoff wall bottom opening sizes (S) (S = 2 cm, 4 cm, 6 cm and 8 cm) and inclination angles (θ = 0°, 15°, 30° and 45°) in sloping beach coastal aquifers. The mixing zone width (W_2_) at average saturated death shows a sharp initial increase over the first half hour in all slope scenarios. For higher slopes, the breadth peaks earlier. The value of W_2_ rises from roughly 1.5 cm to roughly 3.0 cm for θ = 0^o^ and from 1.5 cm to roughly 4.5 cm for θ = 45^o^, signifying a roughly 50% increase in discharge width for the highest slope initially. W_2_ exhibits a modest fall after the initial surge, but it stabilises after around 80 min. For θ = 0^o^, 15^o^, 30^o^ and 45^o^, the steady state W_2_ values at the end of the simulation (180 min) are roughly 2.6 cm, 3.2 cm, 3.5 cm and 3.8 cm, respectively, with a statistically significant increasing trend with slope angle. When SWI into the freshwater aquifer causes the mixing zone to expand, the mixing zone width (W_2_) first rises over time. This is followed by a more gradual increase, until a steady state is eventually reached. While the slower growth in later time periods indicates that the system is stabilising and finding equilibrium between the freshwater and seawater interaction, the first quick increase in mixing zone width is predicted as seawater infiltrates the aquifer. Figure 6 illustrates how the SWI process progressively slows down as the mixing zone width stabilises. Over time, the mixing zone’s growth becomes less noticeable, especially as the cutoff wall’s influence on limiting its spread grows stronger. The rate at which the mixing zone width increases over time is significantly influenced by the inclination angle (θ). Because the flow path is more direct at θ = 0° (vertical beach aquifer), the mixing zone width grows the fastest. In the case of increasing inclination degrees (15°, 30° and 45°), the width of the mixing zone rises. The hydraulic gradients become more complicated in a steeper aquifer, which slow the progression of SWI and causes the mixing zone to grow more gradually. The more complicated flow dynamics in these situations impede quick SWI, delaying the effectiveness of the cutoff wall. As seen in the figure, the rate of mixing zone expansion decreases as the inclination angle increases, especially for greater values of θ (such as 45°).

Smaller Bottom Openings (S = 2 cm, 4 cm): The width of the mixing zone grows more slowly over time when the cutoff wall has a smaller bottom opening. Compared to larger apertures, the mixing zone width stabilises earlier because the lower bottom opening size offers a higher resistance to SWI. This outcome supports the finding by Chang et al.^44^ that the cutoff wall’s narrower apertures are better at regulating SWI and preventing the mixing zone from expanding. Larger Bottom Openings (S = 6 cm, 8 cm, and 10 cm): When the bottom opening is larger, more saltwater can flow through the hole before the barrier begins to affect the invasion, which causes the mixing zone width to initially rise more quickly. Figure 6 indicates the temporal evolution of the width of the mixing zone (W_2_) under different four inclination angles (0°, 15°, 30°, and 45°) for six tested conditions (a-f). In all tested cases, (W_2_) increased rapidly during the initial time of simulation 20–40 min, reached a maximum value, and then gradually declined toward a steady state condition. The impact of the inclination angle beach face became more pronounced from Fig. 6a and f. In Fig. 6a, the maximum values of (W_2_) increased from approximately 3.1 cm at 0° to 4.2 cm at 45°, corresponding to an increase of about 35%. Similarly, in case of S=10 cm, the peak width increased from approximately 4.3 cm to 5.6 cm, indicating an increase of nearly 30%. Greater differences were observed in the cases S = 8 cm and S = 6 cm, where the maximum value of (W_2_) at 45° exceeded that at 0° by approximately 1.5–2.0 cm (30–40%). The largest variation occurred in S = 4 cm and S = 2 cm, where the peak of the width of the mixing zone (W_2_) increased from about 6.2–6.5 cm at 0° to approximately 8.0–9.2 cm at 45°, corresponding to increases of nearly 30–45%. At the end of the simulation time (180 min), the residual (W_2_) under 45° remained approximately 0.8–2.0 cm higher than that under 0°, depending on the tested inclination angle and opening below cutoff wall conditions. These results quantitatively illustrate that increasing the inclination angle of beach coastal face enhanced lateral seawater intrusion wedge expansion and delayed its subsequent reduction, with the magnitude of this effect becoming progressively stronger were using the cutoff wall with higher depths with small bottom opening size. Over time, however, the cutoff wall still plays a role in reducing the mixing zone width, but this effect is more gradual than for smaller openings.

### **Hydrodynamic behaviour of the area of the mixing zone (A**_**m**_**) at average saturated depth in sloping beach coastal aquifers**

**Fig. 7:**
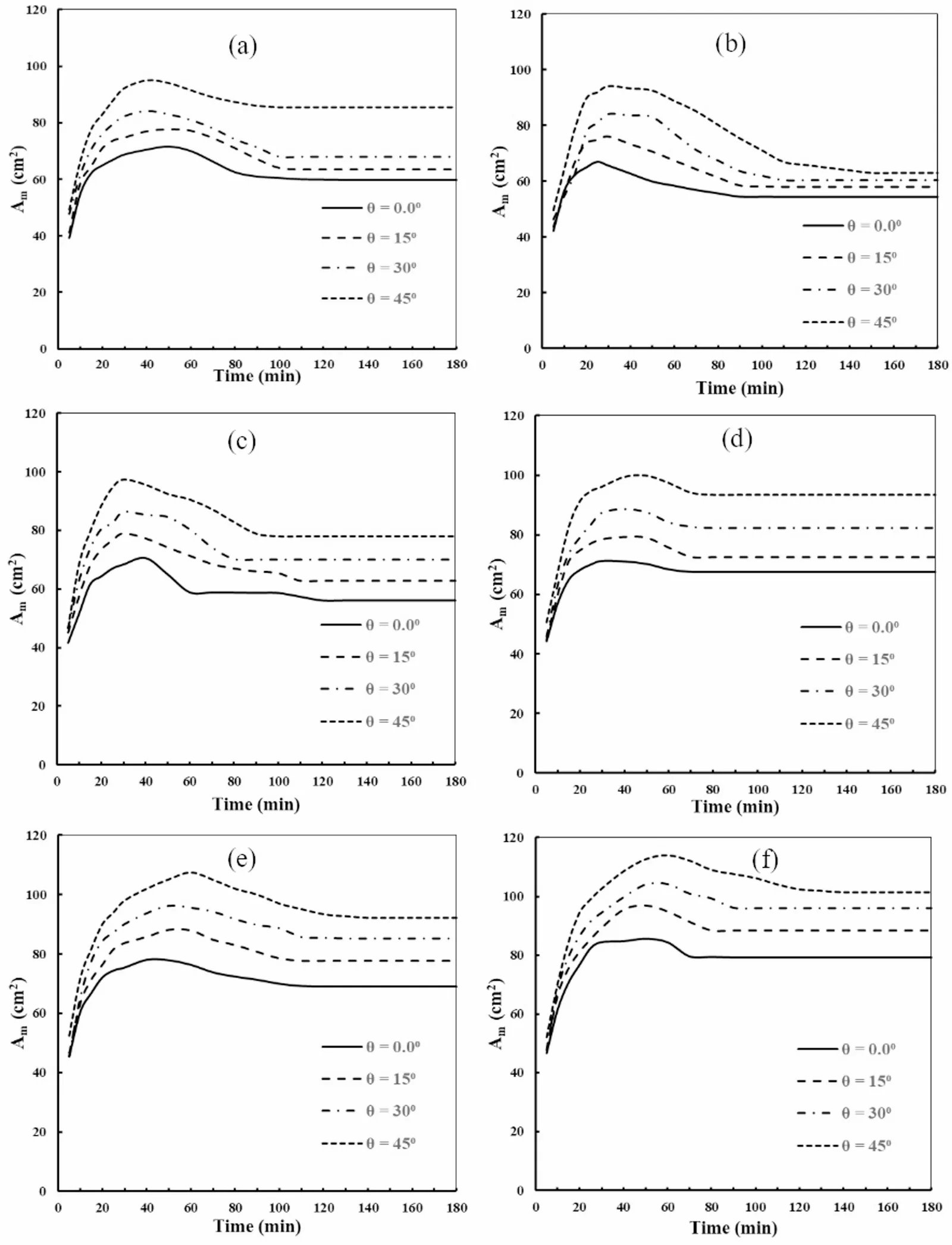
Hydrodynamic behaviour of the area of the mixing zone (A_m_) for different values of sloping beach coastal aquifers θ = 0.0, 15, 30 and 45 for different cases of cutoff: (a) No cutoff, (b) S = 10 cm, (c) S = 8 cm, (d) 6 cm, (e) 4 cm and (f) 2 cm.

The hydrodynamic behaviour of the area of the mixing zone (A_m_) in sloping beach coastal aquifers is shown in Fig. 7 for a range of inclination angles (θ = 0°, 15°, 30° and 45°) and bottom opening sizes (S) (S = 2 cm, 4 cm, 6 cm, 8 cm and 10 cm) of the cutoff wall. Initially, for all slope situations, A_m_ rises quickly over the first half hour, indicating the growth of the freshwater-saltwater mixing interface. There is a positive correlation between slope angle and A_m_ magnitude. In particular, A_m_ increases by about 54% between the lowest and highest slope angles during the early phase, rising from around 40 cm² to 65 cm² for θ = 0^o^ and from 40 cm² to about 100 cm² for θ = 45^o^. Between 40 and 80 min, following the peak, A_m_ shows a modest decrease, and then the area stabilises. The mixing area converges to roughly 58 cm², 72 cm², 80 cm² and 88 cm² for θ = 0^o^, 15^o^, 30^o^ and 45^o^, respectively, during the steady-state period (120–180 min). This suggests that slope has a lasting impact on preserving a wider mixing zone at equilibrium.

As seawater seeps into the freshwater aquifer, the area of the mixing zone (A_m_) grows with time. The mixing zone area then gradually stabilises, becoming more noticeable as the system gets closer to equilibrium. The early stages of SWI are reflected in the mixing zone area (A_m_), which at first rapidly increases. The rate of increase then slows down with time, a sign that the system is approaching equilibrium. The coastal aquifer’s inclination angle (θ) has a major effect on how quickly the mixing zone area grows over time. Because the flow is more direct and seawater may enter more effectively at θ = 0° (vertical beach aquifer), the mixing zone area grows the fastest. The extension of the mixing zone area is immediately constrained by the cutoff wall. The mixing zone area grows more slowly at an increasing degree of inclination (15°, 30° and 45°). The area of the mixing zone grows more slowly in steeper aquifers due to changed hydraulic gradients and more complicated flow dynamics. In steeper aquifers, the cutoff wall’s effect is less immediate, as seen in the figure.

These results are consistent with the mentioned published article, which also noted that the impact of physical barriers is more gradual, and that seawater invasion is slower in steeper aquifers. The aquifer slope affects the dynamics of SWI and the development of the mixing zone, causing the increasing inclination angle to cause a slower rise in the mixing zone area. Smaller Bottom Openings (S = 2 cm, 4 cm): In these instances, the area of the mixing zone increases more gradually, and the impact of the cutoff wall becomes more noticeable early on. The mixing zone area stabilises more quickly because the narrower bottom apertures better prevent seawater invasion. The smaller bottom openings led to a quicker response in restricting the expansion of the mixing zone. Greater Bottom Openings (S = 6 cm, 8 cm and 10 cm): At first, the area of the mixing zone grows more slowly. More seawater can enter through larger apertures before the barrier starts to affect the mixing zone. Figure 7 illustrates that the area of the seawater-freshwater mixing zone (A_m_) increased rapidly during the early stage of the simulation, reached a maximum value, and then gradually stabilized. Increasing the inclination angle of beach coastal face from 0° to 45° consistently enlarged the seawater-freshwater mixing zone (A_m_) across all tested cases, with peak (A_m_) increasing from approximately 70–85 cm² at 0° to 95–115 cm² at 45°, corresponding to an increase of about 25–40%. The influence of inclination angle became more evident from Fig. 7a and f, where higher angles maintained larger seawater-freshwater mixing zone (A_m_) throughout the simulation time. At 180 min, seawater-freshwater mixing zone (A_m_) under 45° remained approximately 15–25 cm² greater than that observed at 0°, indicating enhanced seawater intrusion under steeper inclinations. In contrast to smaller openings, this effect occurs more gradually, supporting the finding of Chang et al.^44^ that larger openings cause the mixing zone to stabilise more slowly.

### **Hydrodynamic behaviour of the seawater wedge’s length of 90% isohaline (L**_**90%**_**)** in **sloping beach coastal aquifers**

**Fig. 8:**
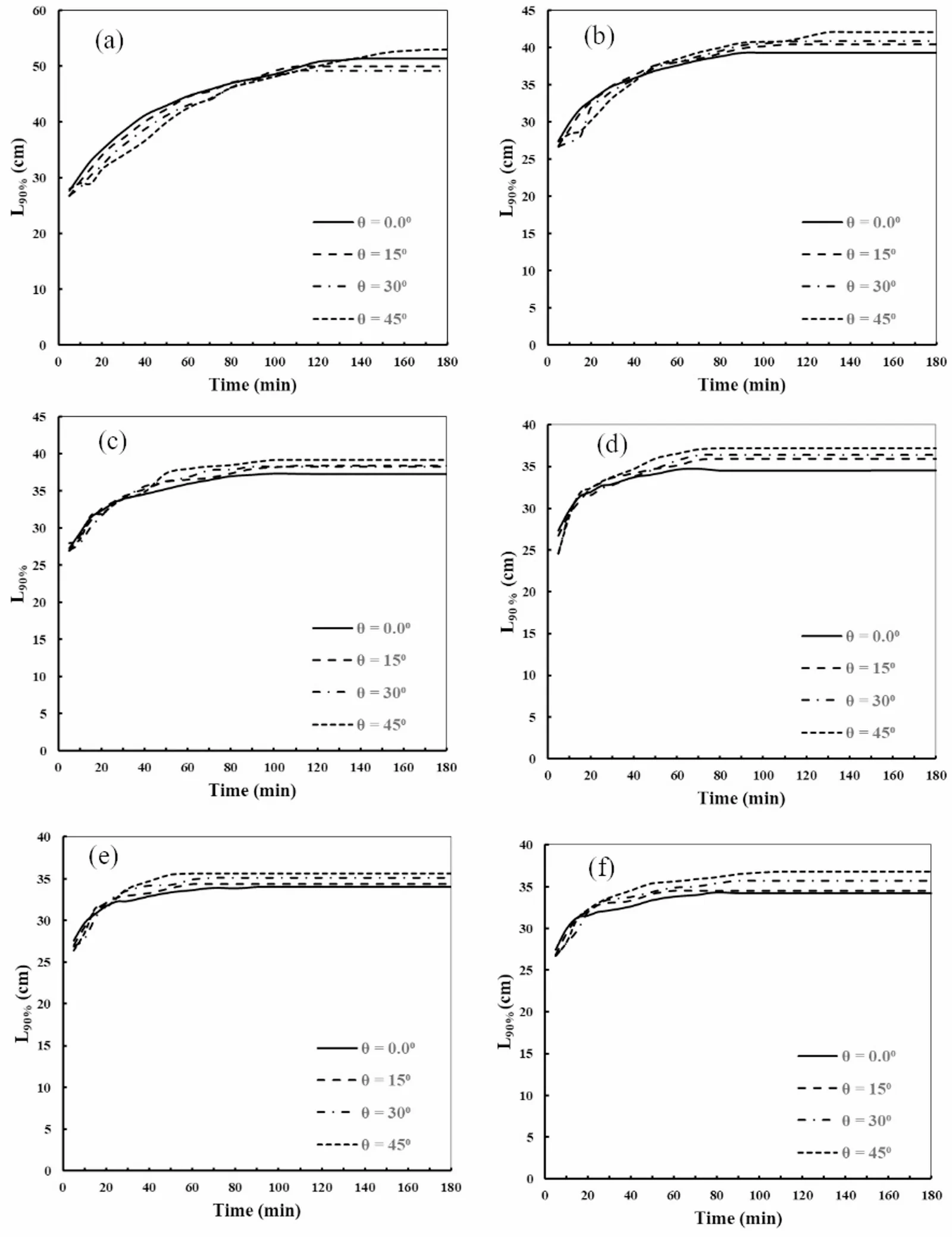
Hydrodynamic behaviour of the length of the seawater wedge (L_90%_) for different values of sloping beach coastal aquifers θ = 0.0, 15, 30 and 45 for different cases of cutoff: (a) No cutoff, (b) S = 10 cm, (c) S = 8 cm, (d) 6 cm, (e) 4 cm and (f) 2 cm.

The hydrodynamic behaviour of the seawater wedge’s length of 90% isohaline (L_90%_) over time is depicted in Fig. 8 for a range of cutoff wall bottom opening sizes (S) (S = 2 cm, 4 cm, 6 cm, 8 cm and 10 cm) and inclination angles (θ = 0°, 15°, 30° and 45°) in sloping beach coastal aquifers. In all slope configurations, L_90%_ steadily rises with time, indicating the saltwater front’s slow onshore movement. In contrast to the (θ = 0°) slope, steeper slopes first show somewhat lower L_90%_ values from 0 to about 60 min, indicating a delayed progression of high saline levels in steeper profiles. At 60 min, for instance, L_90%_ reaches about 42 cm, while for steeper slopes, it stays around 38–40 cm. With only slight variations seen near the end of the simulation, the L_90%_ values for all slopes converge as the run time exceeds 100 min. For θ = 0^o^, L_90%_ stabilises at 52 cm at 180 min, while the other slopes converge slightly below, ranging between 48 and 50 cm, demonstrating a difference of less than approximately 8% in all cases. As seawater seeps into the freshwater aquifer, the seawater wedge length (L_90%_) gradually grows. The early stage of seawater penetration is shown in the wedge length’s initial rapid growth. The growth slows over time, demonstrating the impact of the cutoff wall as a physical barrier in preventing additional seawater invasion. As seawater seeps into the aquifer in the early phases, L_90%_ then rises swiftly. However, the rate of rise eventually slows down and stabilises, which is in line with the findings of Chang et al.^44^. According to their study, as the cutoff wall starts to restrict the seawater wedge’s movement, seawater invasion advances more slowly.

The pace of the seawater wedge’s growth over time is significantly influenced by the coastal aquifer’s inclination angle (θ): Since the flow is more consistent and seawater may enter effectively at θ = 0° (vertical beach aquifer), the seawater wedge length grows the fastest there. The seawater wedge’s growth rate falls as the inclination angle rises (15°, 30° and 45°). The flow dynamics become more complicated, and the seawater wedge moves more slowly in aquifers that are steeper. The effect of the slope on hydraulic gradients is reflected in this behaviour. In steeper aquifers, the length of the seawater wedge increases more slowly, and the cutoff wall’s action is more delayed. Steeper aquifers cause slower intrusion over time, as evidenced by the decreasing rate of the seawater wedge’s expansion as the inclination angle increases. Smaller Bottom Openings (S = 2 cm, 4 cm): Because a smaller opening size offers a stronger barrier against SWI, the seawater wedge length initially rises more slowly for smaller openings. Early in the simulation, the impact of the cutoff wall becomes more noticeable, which causes the seawater wedge to stabilise more quickly in the early phases. The seawater wedge length rises more slowly at larger bottom openings (S = 6 cm, 8 cm, and 10 cm), as more seawater can pass through the wider gaps before the barrier stops additional passage. Because more seawater can enter before the physical barrier starts to slow down the incursion, the cutoff wall’s initial impact on the seawater wedge length is delayed. Figure 8 displays that the seawater intrusion wedge length (L_90%_) increased progressively with time and approached a nearly steady state value after approximately 80–120 min. Unlike the seawater intrusion wedge area and seawater-freshwater mixing zone, the impact of inclination angle on (L_90%_) was relatively limited, with all tested simulations exhibiting similar growth trends. Increasing the inclination angle from 0° to 45° produced only a modest increase in the final seawater intrusion wedge length (L_90%_), generally less than 5–10% across the six tested conditions. At 180 min, the differences among inclination angles remained small, indicating that inclination had a stronger impact on lateral seawater intrusion than on advancement of seawater intrusion wedge length.

## Hydrodynamic behaviour of the average mixing zone width (MZW) in sloping beach coastal aquifers

**Fig. 9:**
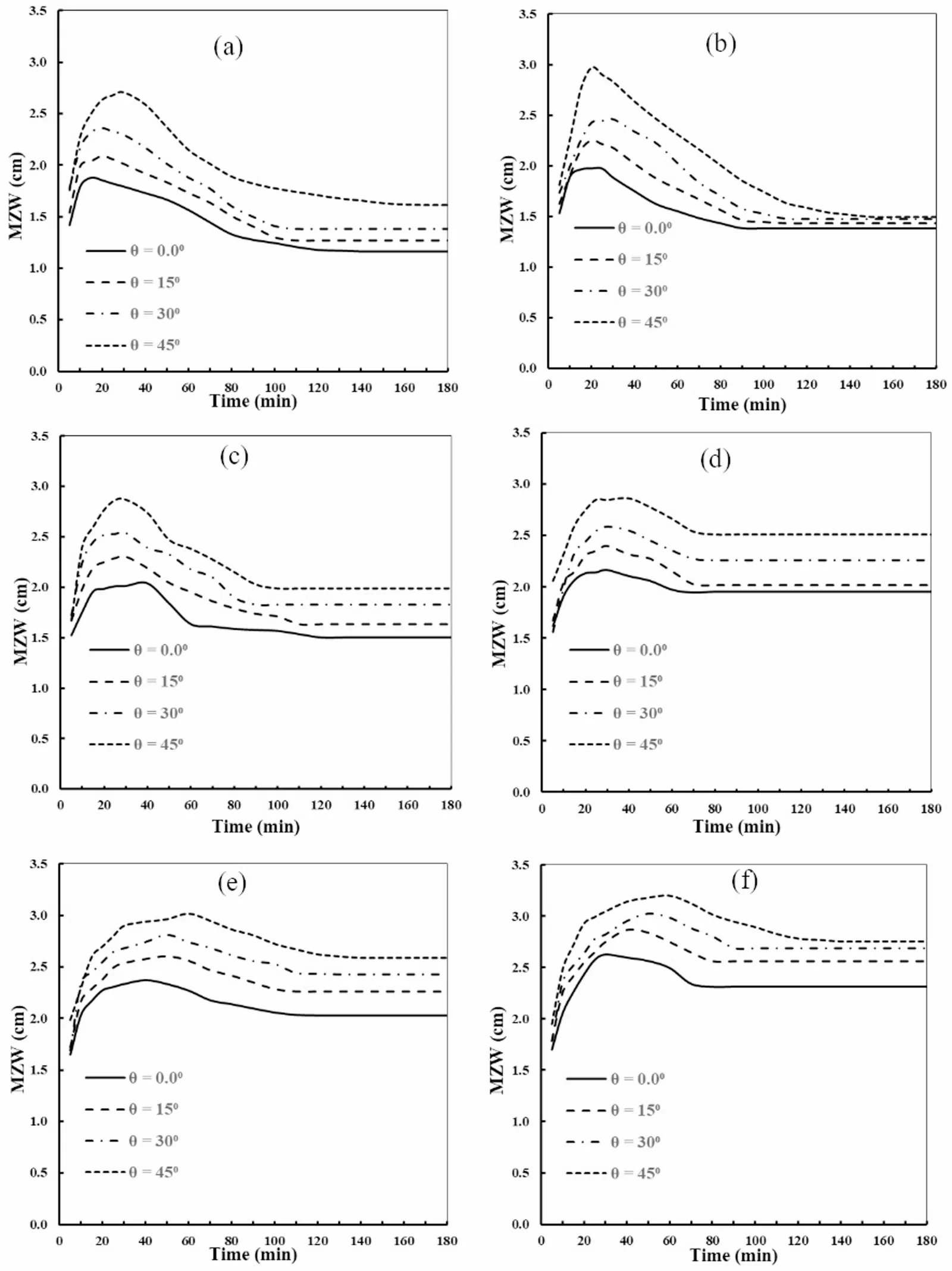
Hydrodynamic behaviour of the average mixing zone width (MZW) for different values of sloping beach coastal aquifers θ = 0.0, 15, 30 and 45 for different cases of the cutoff wall opening: (a) No cutoff, (b) S = 10 cm, (c) S = 8 cm, (d) 6 cm, (e) 4 cm, and (f) 2 cm.

The hydrodynamic behaviour of the average mixing zone width (MZW) in sloping beach coastal aquifers is shown in Fig. 9 for a range of inclination angles (θ = 0°, 15°, 30° and 45°) and bottom opening sizes (S) (S = 2 cm, 4 cm, 6 cm, 8 cm and 10 cm) of the cutoff wall. The average mixing zone width (MZW) initially rises quickly, peaking for all slope angles during the first half hour. The peak MZW shows a positive correlation with the beach slope, increasing by around 66% with slope steepness, from about 1.8 cm for θ = 0^o^ to nearly 3.0 cm for θ = 45^o^. The MZW peaks and then steadily drops for about an hour before levelling off. In addition, the MZW stabilises at lower levels throughout the steady state phase (120–180 min), around 1.2 cm, 1.4 cm, 1.5 cm and 1.7 cm for θ = 0^o^, 15^o^, 30^o^ and 45^o^, respectively. Steeper slopes continually maintain a wider mixing zone than flatter slopes, even though the values are reduced from peak values. As the mixing zone forms and SWI into the freshwater aquifer occurs, the average mixing zone width (MZW) first rises quickly over time. The pace of increase slows down, and the mixing zone width stabilises with time. When the saltwater wedge approaches the barrier, the impact of the cutoff wall on the mixing zone becomes more noticeable. This is depicted in the picture: as the mixing zone breadth increases more slowly over time, the cutoff wall progressively stops seawater flow, stabilising the area. The pace at which the mixing zone width expands over time is strongly influenced by the inclination angle (θ): Due to the effective horizontal movement of seawater through the aquifer, the mixing zone width increases most quickly when θ = 0° (vertical beach aquifer). The rise in mixing zone width is immediately and significantly slowed down by the cutoff wall. The rate at which the width of the mixing zone increases decreases as the inclination angle increases (15°, 30°, and 45°). The development of SWI and mixing zone growth is slowed down by steeper aquifers because they produce more intricate flow dynamics. As the aquifer’s slope rises, the barrier’s effect becomes more gradual, as seen in the figure.

Smaller Bottom apertures (S = 2 cm, 4 cm): In these situations, the cutoff wall may more successfully prevent SWI due to the smaller apertures, which causes the mixing zone width to rise more gradually. The impact of the cutoff wall is seen sooner, causing the mixing zone width to stabilise more quickly. According to Chang et al.^44^, smaller bottom openings offer superior resistance to SWI, and this is consistent with the smaller apertures’ ability to allow more instant control over the mixing zone. Larger Bottom apertures (S = 6 cm, 8 cm, and 10 cm): In these circumstances, the mixing zone width increases more slowly at first because more seawater can enter through the larger apertures before the barrier begins to drastically restrict it. As a result, the impact of the cutoff wall gets more gradual, and the mixing zone keeps expanding for a longer time. Figure 9 displays the variation of mixing zone width (MZW) with time (0–180 min) at inclination angles of 0°, 15°, 30°, and 45°. In all cases, MZW increased rapidly during the initial 20–60 min, reached a maximum value, and then gradually declined or stabilized. Higher inclination angles consistently produced greater MZW values, with 45° viewing the highest response and 0° the lowest. In Fig. 9 (a–c), peak MZW values ranged from approximately 2.0–3.0 cm, followed by a decrease to 1.2–2.0 cm at 180 min. Figure 9(d) exhibited a rapid rise and maintained relatively stable values between 1.9 and 2.4 cm. The highest MZW levels were observed in Figs. 9(e) and 9(f), where peak values reached approximately 3.0–3.2 cm at 45° and stabilized between 2.0 and 2.8 cm during the later stages. Overall, increasing the inclination angle from 0° to 45° enhanced both peak and steady-state MZW values by roughly 20–50%, indicating a strong positive effect of inclination angle on MZW development throughout the tested simulation period.

The simulations show that seawater intrusion is controlled by dispersivity, hydraulic conductivity, and cutoff-wall position (Figs. A11, A12, A13, A14, A15, A16 and A17). Increasing longitudinal dispersivity extends saline water inland and produces a wider, smoother mixing zone (Fig. A11), whereas higher transverse dispersivity primarily thickens the interface through crossflow mixing without greatly extending intrusion length (Fig. A12). Lower hydraulic conductivity restricts groundwater movement, resulting in slower, shorter, and more compact seawater intrusion with sharper salinity gradients (Fig. A13 and Fig. A14). Cutoff walls provide increasing protection as they are moved closer to the shoreline (Fig. A15, Fig. A16 and Fig. A17). The 0.25X_c_ position most effectively limits saline wedge development, preserves inland freshwater, and maintains the smallest and sharpest mixing zone across tested configurations.

## Groundwater velocity plot

**Fig. 10:**
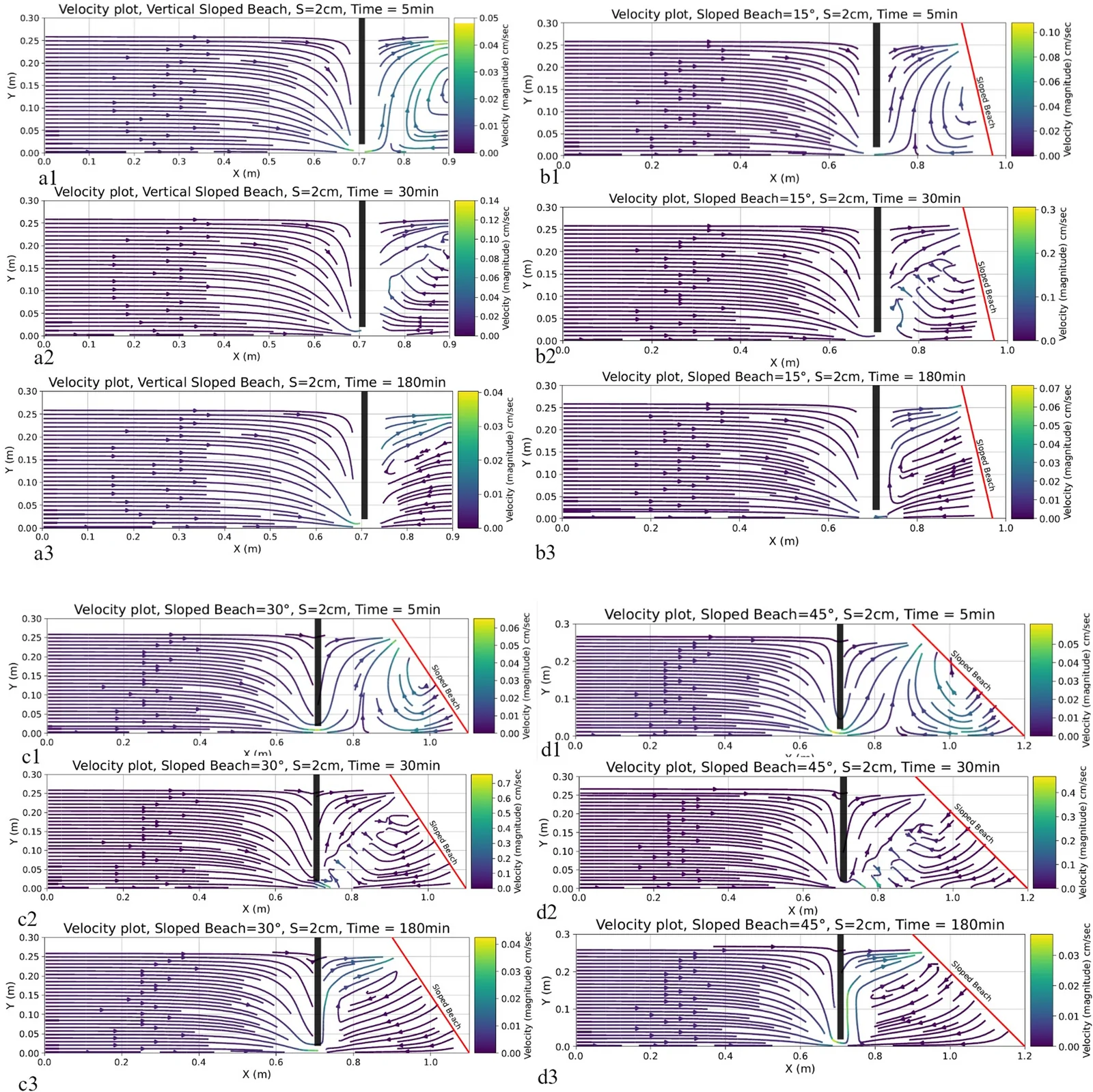
Velocity plot for various sloped beach coastal aquifers: (a) vertical, (b) 15^o^, (c) 30^o^, and (d) 45^o^ at various times equals 5, 30, and 180 min with below cutoff wall opening size S = 2 cm.

Figure 10 demonstrates the evolution of groundwater velocity streamlines in coastal aquifers with beach face slopes of 0° (vertical), 15°, 30°, and 45° for a cutoff wall opening of S = 2 cm at selected time are 5, 30, and 180 min. The groundwater flow is mainly directed from the freshwater inland boundary toward seawater boundary, where streamlines converge and bend around the cutoff wall structure location. The color contours characterize the groundwater velocity magnitude and highlight zones of accelerated groundwater flow. At 5 min, the groundwater velocity values are generally low, ranging from approximately 0.0 to 0.05 cm/sec for all sloping beach face shapes. Higher groundwater velocities are concentrated close to the outlet exit face and along the seaward boundary, where the groundwater flow begins to adjust to the imposed hydraulic conditions. Small recirculation cells are visible on the seawater boundary side, predominantly for the inclined sloping beaches cases. At 30 min, the groundwater flow system develops more active and distinct circulation zones develop. Maximum groundwater velocities increase to approximately 0.14 cm/sec for the vertical coastal beach case, 0.30 cm/sec for the 15° sloping beach, 0.70 cm/sec for the 30° sloping beach, and 0.60 cm/sec for the 45° sloping beach. These peak groundwater velocity values occur primarily around the cutoff wall bottom opening and within the recirculation regions close to seawater boundary, representing intensified groundwater flow concentration and seawater-freshwater mixing. Increasing sloping beach inclination angle results in stronger streamline curvature and greater flow deflection toward the sloping boundary.

By 180 min, the groundwater velocity field approaches a more stable condition. Velocity magnitudes decline to approximately 0.0–0.04 cm/sec for the vertical sloping beach case, 0.0–0.07 cm/sec for the 15° sloping beach, 0.0–0.04 cm/sec for the 30° sloping beach, and 0.0–0.03 cm/sec for the 45° sloping beach. Although the overall flow weakens, circulation patterns remain evident near the outlet seawater boundary. The results prove that beach slope inclination angle significantly controls the groundwater flow paths, with steeper slopes creating stronger transient velocities and more pronounced recirculation throughout the intermediate stage of seawater intrusion system development.

Figure A18 illustrates the groundwater velocity field for coastal aquifers with inclination angle beach slopes of 0°, 15°, 30°, and 45° and bottom cutoff wall opening of S = 4 cm at 5, 30, and 180 min. The streamline patterns show that groundwater generally flows from the freshwater inland side toward the outlet seawater boundary, where flow convergence occurs near the bottom cutoff wall opening. At 5 min, groundwater flow velocity magnitudes are relatively low, ranging from approximately 0.0–0.05 cm/sec for all tested slope beach configurations, with localized acceleration near the outlet seawater boundary and along the sloping beach face. Small circulation cells are noticeable on the seaward boundary side, reflecting the initial adjustment of the flow system. At 30 min, the groundwater velocity field becomes more dynamic, with maximum values increasing substantially. Peak groundwater flow velocities reach about 0.30 cm/sec for the vertical and 15° beaches, 0.60 cm/sec for the 30° sloping beach, and 0.50 cm/sec for the 45° sloping beach. These elevated groundwater velocities are concentrated around the bottom cutoff wall opening and within the recirculation zones, where strong hydraulic gradients promote accelerated groundwater flow. Streamlines also exhibit greater curvature as beach inclination increases, demonstrating enhanced interaction between the groundwater aquifer flow and the sloping seawater boundary.

By 180 min, the groundwater flow field evolves toward steady-state condition, and groundwater velocity magnitudes decline to approximately 0.0–0.05 cm/sec for the vertical and 15° sloping beaches, 0.0–0.06 cm/sec for the 30° sloping beach, and 0.0–0.04 cm/sec for the 45° sloping beach. While groundwater velocities decline, recirculation zones remain evident near the outlet seawater boundary region. Overall, the results demonstrate that inclination angle of beach face strongly impacts the groundwater circulation and groundwater velocity distribution, with steeper slopes generating more pronounced flow deflection, stronger recirculation, and higher transient peak groundwater velocities, particularly during the intermediate stage of flow development.

In summary, the results quantitatively demonstrate that aquifer inclination significantly modifies seawater intrusion dynamics, particularly during transient conditions. Steeper slopes enhance early-time SGD, enlarge mixing zone dimensions (W_1_, W_2_, MZW, and A_m_), and increase intrusion wedge length (T_L_), while their influence weakens for some parameters (SGD and L_90%_) at steady state. The interaction between aquifer slope and cutoff wall geometry further amplifies these effects, especially under small bottom opening conditions.

## Conclusion

This study provides a comprehensive quantitative assessment of how coastal aquifer inclination angle (0°, 15°, 30°, and 45°) affects seawater intrusion dynamics under various cutoff wall configurations. The analysis of SGD confirms that aquifer inclination angle strongly controls early-time flow behaviour. Increasing the slope angle from 0° to 45° rises the initial SGD by approximately 20–40%, with a maximum increase of around 31% (from 4.8 × 10^− 4^ to 6.3 × 10^− 4^ m³/min). However, this influence diminishes over time, and all cases converge toward similar steady-state values (1.1 × 10^− 4^ to 3.1 × 10^− 4^ m³/min), with differences reduction to less than 5%. This shows that slope angle influences are predominant during transient conditions but become negligible at steady state condition. The seawater intrusion wedge length (T_L_) exhibits a persistent and strongly slope-dependent response. Final T_L_ values increase substantially with inclination, rising from approximately 43 cm at 0° to about 67 cm at 45°, indicating an increase of approximately 55–56%. Depending on cutoff wall conditions, increases between 55% and 150% were observed across scenarios. Unlike SGD, the impact of slope angle on T_L_ remains pronounced even at steady state, confirming that aquifer inclination angle has a lasting impact on inland seawater intrusion extent.

Mixing zone geometry is also significantly influenced by slope inclination angle. The bottom mixing zone width (W_1_) declines rapidly with time in all tested cases, with the strongest reductions observed at higher angle slopes. For example, at 45°, W_1_ reduces by approximately 80–90%, from around 8.3–8.5 cm to 0.8–1.7 cm. On the other hand, the 0° case starts at lower values (2.5–3.0 cm) and converges to similar final conditions (0.7–1.4 cm), indicating that slope angle mainly controls the early-stage mixing structure rather than long-term steady state behaviour. The central mixing zone width (W_2_) demonstrates a non-monotonic temporal evolution, increasing initially, reaching a peak, and then gradually declining. Higher slopes consistently amplify peak values, with increases of approximately 30–45% observed between 0° and 45°, particularly under smaller bottom opening conditions of cutoff wall. Residual differences at 180 min remain significant (up to 0.82 cm), confirming a sustained impact of slope angle on lateral seawater intrusion spreading.

Similarly, the total mixing zone area (A_m_) increases with slope angles across all cases. Peak values rise from around 70–85 cm² at 0° to 95–115 cm² at 45°, corresponding to an increase of approximately 25–40%. Even at the end of the simulation, the 45° case maintains a larger mixing zone area (by 15–25 cm²), demonstrating enhanced and persistent seawater-freshwater interaction in steeper coastal aquifers. The seawater intrusion wedge length defined at 90% salinity (L_90%_) shows comparatively limited sensitivity to slope, with variations generally within 5–10% across all tested cases. This indicates that while aquifer inclination strongly influences lateral dispersion and seawater-freshwater mixing characteristics, its effect on deeper seawater intrusion penetration is relatively minor under the present boundary conditions. Finally, the seawater-freshwater mixing zone width (MZW) exhibits a strong early-time response to slope, with peak increases of approximately 20–50% when the inclination increases from 0° to 45°. Higher slopes consistently produce larger peak and steady-state values, particularly under smaller cutoff wall opening conditions, confirming a strengthened mixing process in steeper coastal aquifers. These findings provide clear quantitative evidence that both coastal slope and engineering design cutoff wall parameters must be jointly considered when evaluating or controlling seawater intrusion in coastal aquifers.

## Supplementary Information

Below is the link to the electronic supplementary material.

Supplementary Material 1

## Data Availability

All data generated or analysed during this study are included in this published article.
