# Supplementary material for: Hydrodynamic behaviour of the seawater-freshwater mixing zone induced by cutoff wall installation in sloping coastal aquifers
Source: Sci Rep. 2026 Aug 20;16:26176. doi: 10.1038/s41598-026-67038-3 (PMC13493985; doi:10.1038/s41598-026-67038-3)
Supplement: Supplementary file 1 [file 41598_2026_67038_MOESM1_ESM.pdf]

Supplementary File

Hydrodynamic behaviour of the seawater-freshwater mixing zone induced by cutoff wall installation in sloping coastal aquifers

Asaad M. Armanuos*[1], Kristian Micko[2], Marcela Bindzárová Gergeľová [3], and Martina Zeleňáková[4]

[1] Irrigation and Hydraulics Engineering Department, Faculty of Engineering, Tanta University, Tanta, Egypt, Email: asaad.matter@f-eng.tanta.edu.eg
[2] Centre of Digital Technologies in Construction, Faculty of Civil Engineering, Technical University of Kosice, 04200 Kosice, Slovakia, Email: kristian.micko@tuke.sk
[3] Institute of Geodesy, Cartography and Geographical Information Systems, Faculty of Mining, Ecology, Process Control and Geotechnology, Technical University of Kosice, 04200 Kosice, Slovakia; marcela.bindzarova.gergelova@tuke.sk

[4] Institute of Environmental Engineering, Faculty of Civil Engineering, Technical University of Košice, 04200 Košice, Slovakia, Email: martina.zelenakova@tuke.sk

Corresponding author: Asaad M. Armanuos

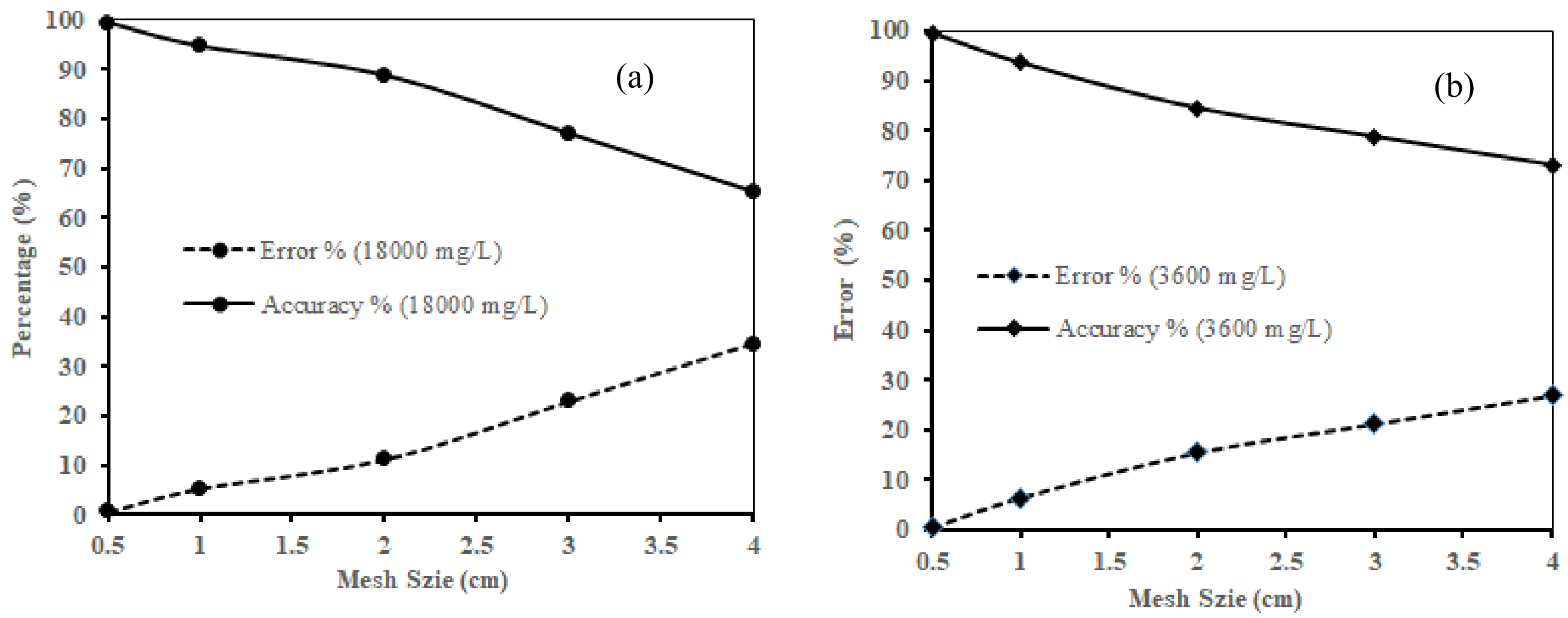

Fig. A1: Computed error and accuracy of SEAWAT code results based on various tested mesh size: (a) concentration line (18,000 mg/L), and (b) concentration line (3,600 mg/L)

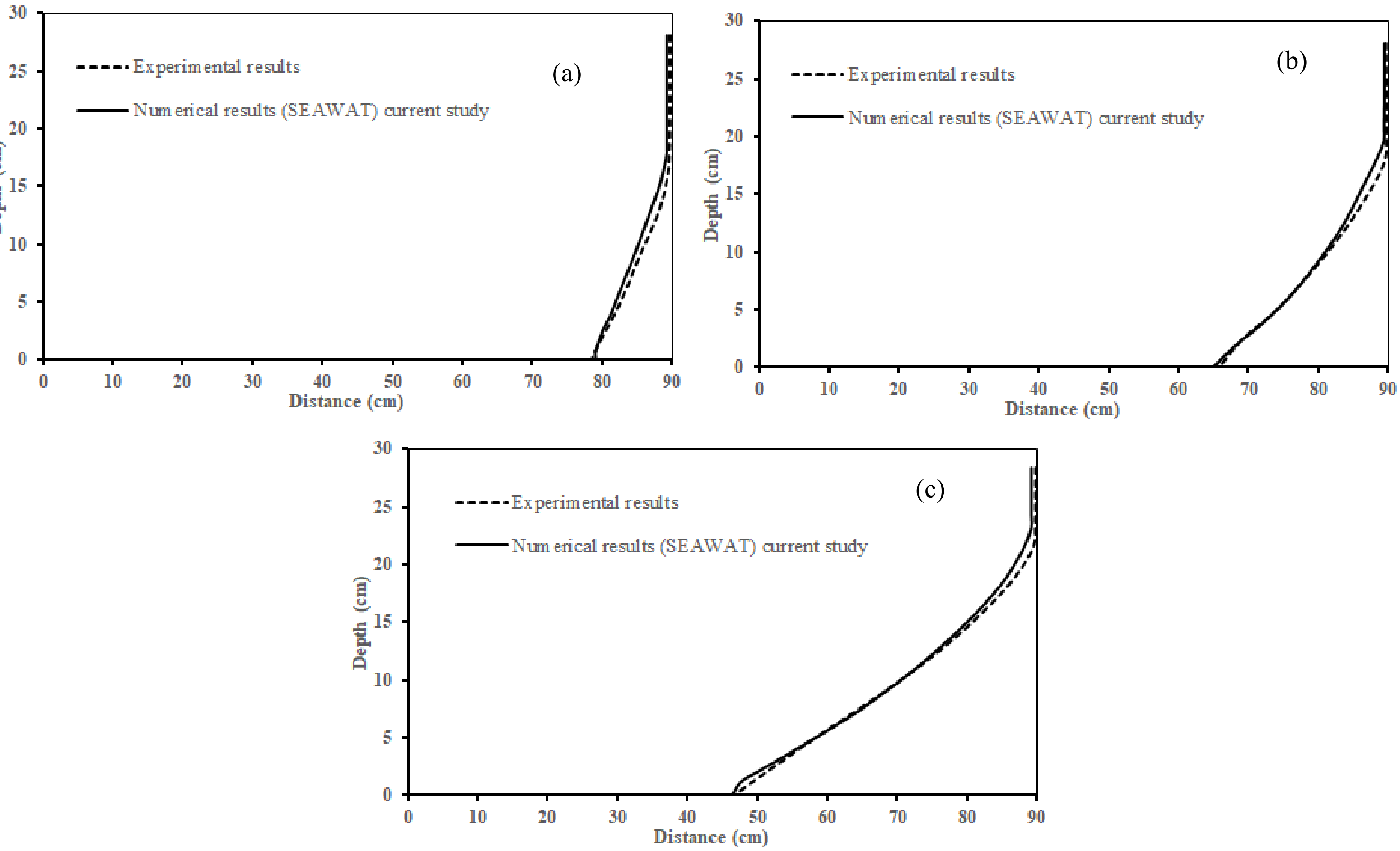

Fig.A2: Comparison between experimental and simulated results of seawater-freshwater interface for concentration contour line (3,6000 mg/L) at various selected time are 5, 30, and 180 min

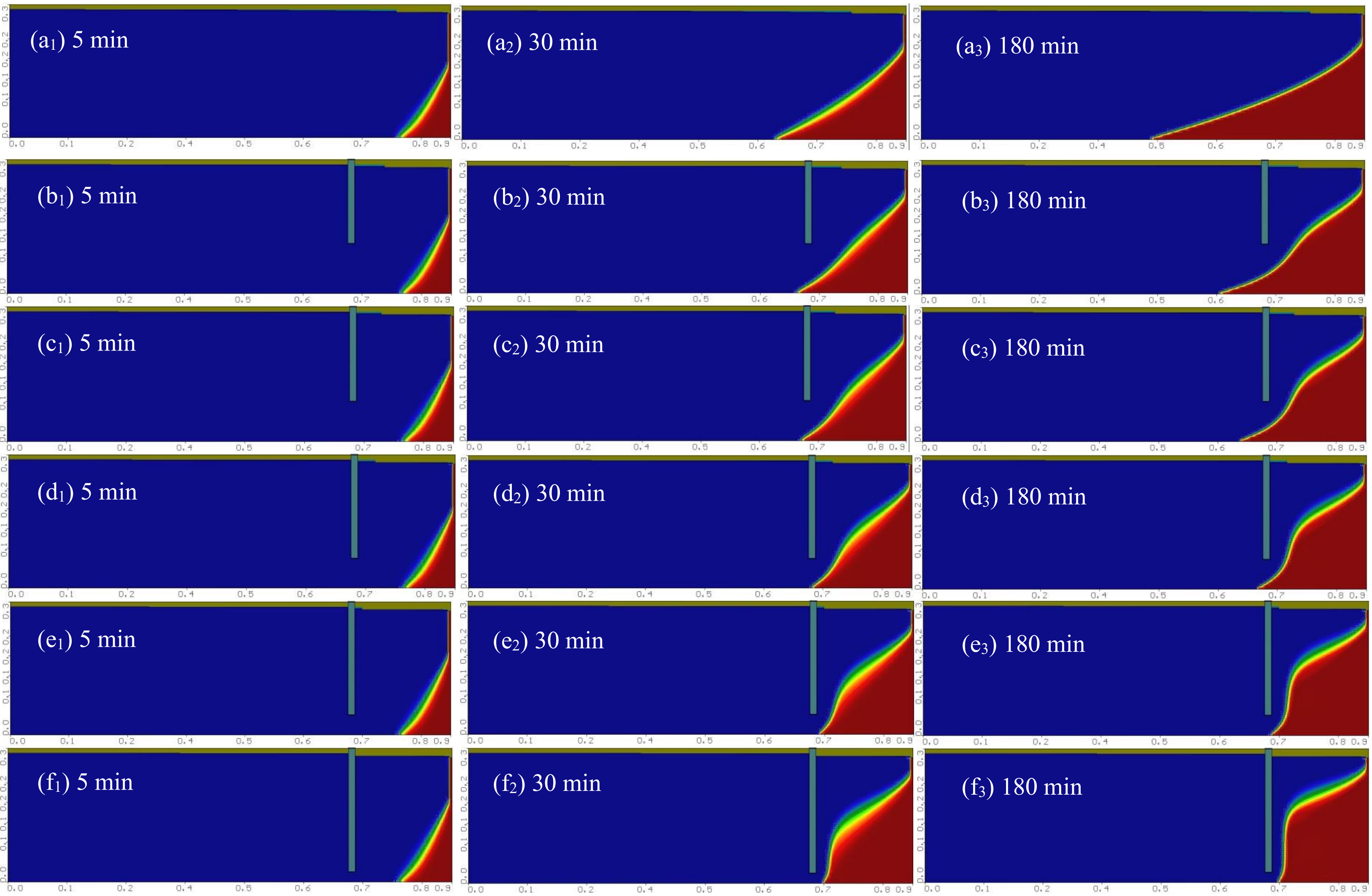

Fig.A3: Steady state seawater intrusion for a vertical slope beach coastal aquifer for (a) without cutoff wall and for different values of space below the cutoff wall S: (b) 10 cm, (b) 8 cm, (b) 6 cm, (e) 4 cm and (f) 2 cm; the black lines indicate the 10, 50 and 90% isohalines of the mixing zone.

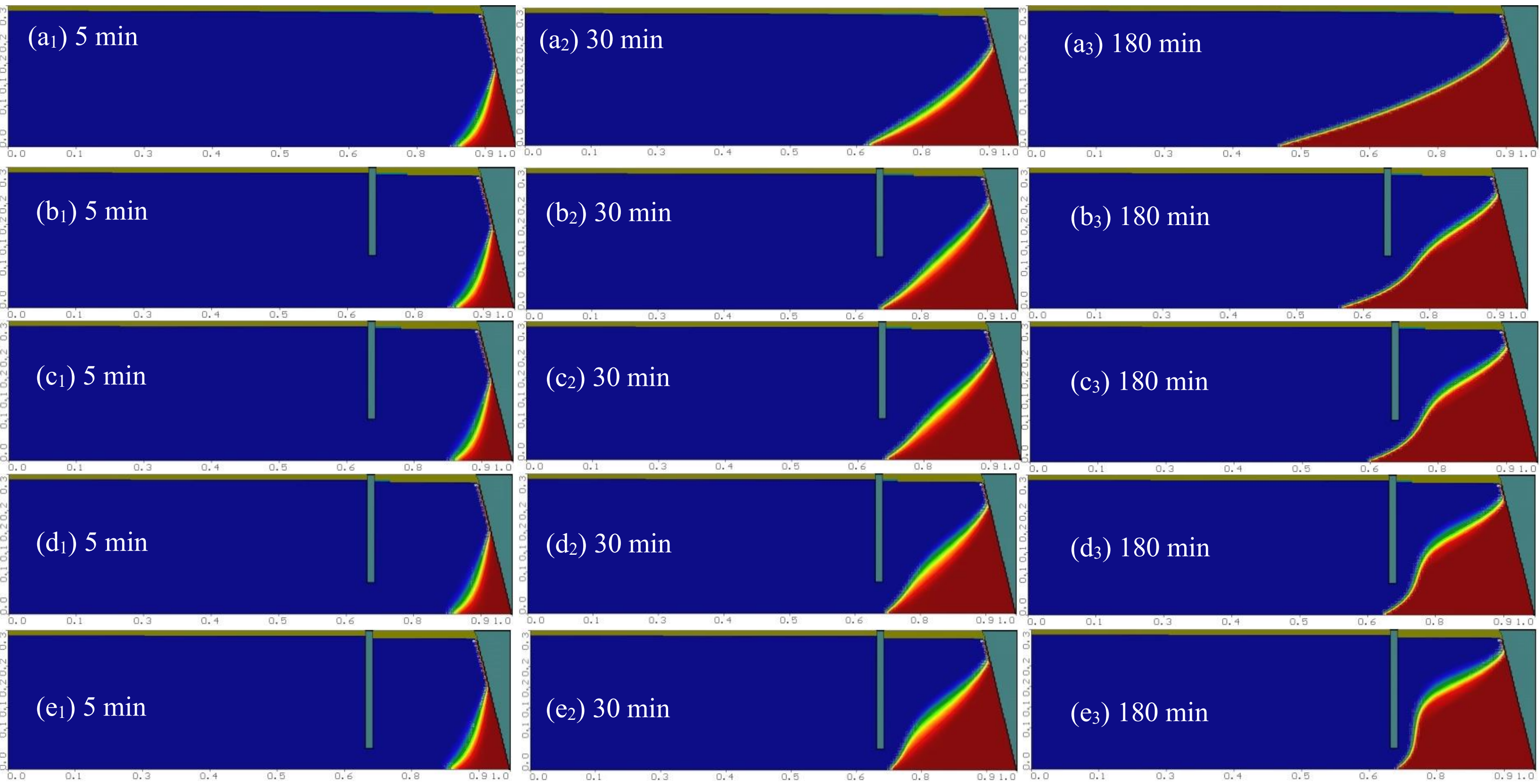

Fig.A4: Steady state seawater intrusion for a sloped beach coastal aquifer with an inclination angle of 15° for (a) without a cutoff wall and for different values of space below the cutoff wall S: (b) 10 cm, (b) 8 cm, (b) 6 cm, (e) 4 cm and (f) 2 cm; the black lines indicate the 10, 50 and 90% isohalines of the mixing zone.

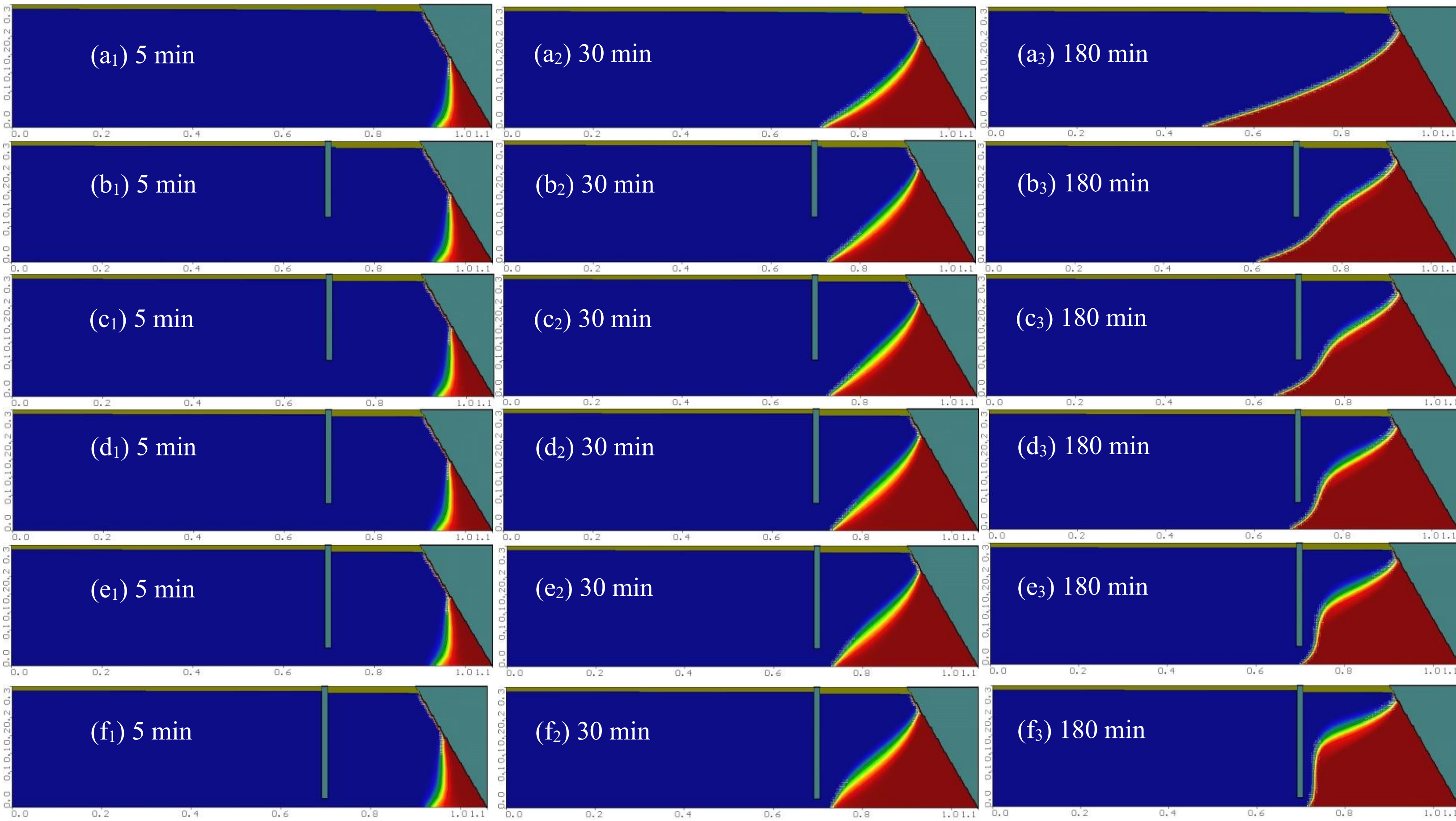

Fig.A5: Steady state seawater intrusion for a sloped beach coastal aquifer with an inclination angle of 30 º for (a) without a cutoff wall and for different values of space below the cutoff wall S: (b) 10 cm, (b) 8 cm, (b) 6 cm, (e) 4 cm and (f) 2 cm; the black lines indicate the 10, 50 and 90% isohalines of the mixing zone.

Hydrodynamic behaviour of the seawater–freshwater mixing zone with varying beach slopes and bottom opening sizes

Fig. A6 shows the development of the seawater–freshwater mixing zone, under the impact of a bottom opening (S) of a cutoff wall, over time for four different coastal beach slope angles: 0°, 15°, 30° and 45°. The progression of isohalines that represent salinity levels of 10% and 90% at various time intervals (5, 30 and 180 minutes) is depicted in each subfigure (a–d), indicating one inclination angle. The active mixing zone is delineated by the region encompassed between the 10% and 90% isohalines, which also acts as a stand-in for evaluating SWI dynamics. In every instance, the mixing zone grows landward over time, representing the slow inland movement of seawater brought on by boundary conditions and density-driven flow. Nevertheless, the opening size S and the coastal slope have a major impact on the geometry and extent of this mixing zone. The mixing zone is comparatively constrained in both vertical and horizontal extents at a beach slope aquifer (0°, Fig. A6a), suggesting limited penetration of inland saltwater. A wider mixing region is produced by raising the slope to 15° (Fig. A6b), which suggests improved lateral dispersion and a longer interface because of higher hydraulic gradients and changed flow routes. At 30° and 45° inclinations (Figs. A6c and A6d, respectively), where the isohalines become steeper and more dispersed, this pattern becomes more noticeable. The mixing zone size is significantly increased, especially around 45°, suggesting that steeper slopes promote more mixing and seawater advances inland.

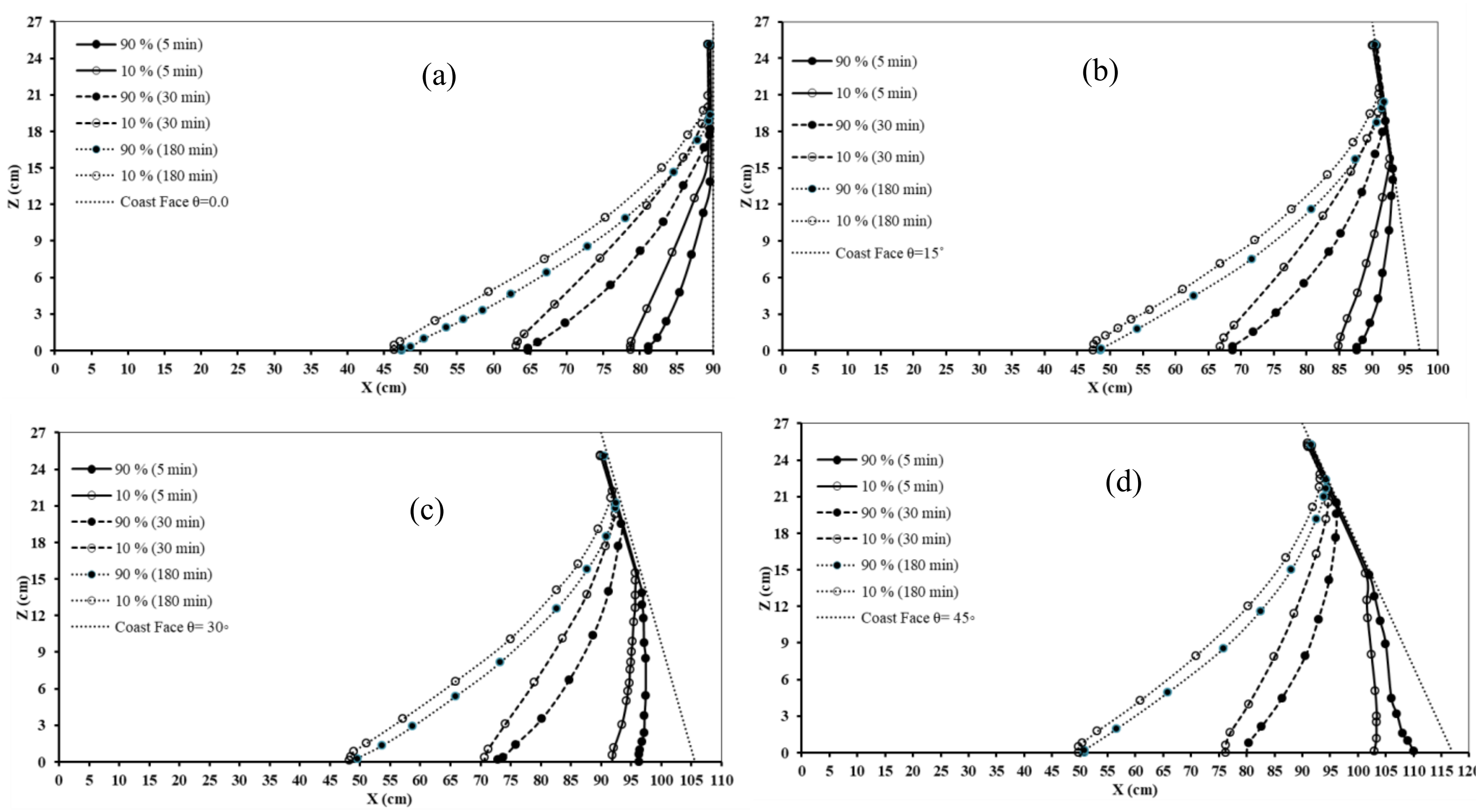

Fig. A6: Hydrodynamic behaviour of the seawater-freshwater mixing zone for different inclination angles of a beach: (a) 0.0°, (b) 15°, (c) 30° and (d) 45°

Hydrodynamic behaviour of the seawater–freshwater mixing zone: influence of bottom opening size below a cutoff wall in a vertical beach coastal aquifer

The temporal evolution of the seawater–freshwater mixing zone for different bottom opening sizes (S) beneath the cutoff wall in a coastal aquifer with a vertical beach slope (0°) is depicted in Fig. A7. The seawater front moves more slowly inland and the mixing zone stays comparatively compact at the maximum bottom opening size (10 cm, Fig. A7a). The mixing zone gradually widens as the bottom opening size drops (8 cm, 6 cm, 4 cm and 2 cm in Figs. A7b–A7e), suggesting a decreased rate of SWI. More freshwater-saltwater interaction is made possible by smaller cutoff openings, which also restrict the seawater front's ability to penetrate inland. The mixing zone is the largest at the smallest bottom opening (2 cm, Fig. A7e), where the seawater front extends farther inland and has a wider lateral spread. This is probably because there is less seawater flow behind the cutoff wall, where the smaller aperture size allows for more effective saltwater dispersion both vertically and horizontally. As the size of the bottom opening decreases, the width of the mixing zone grows; this is especially noticeable with the 2 cm opening.

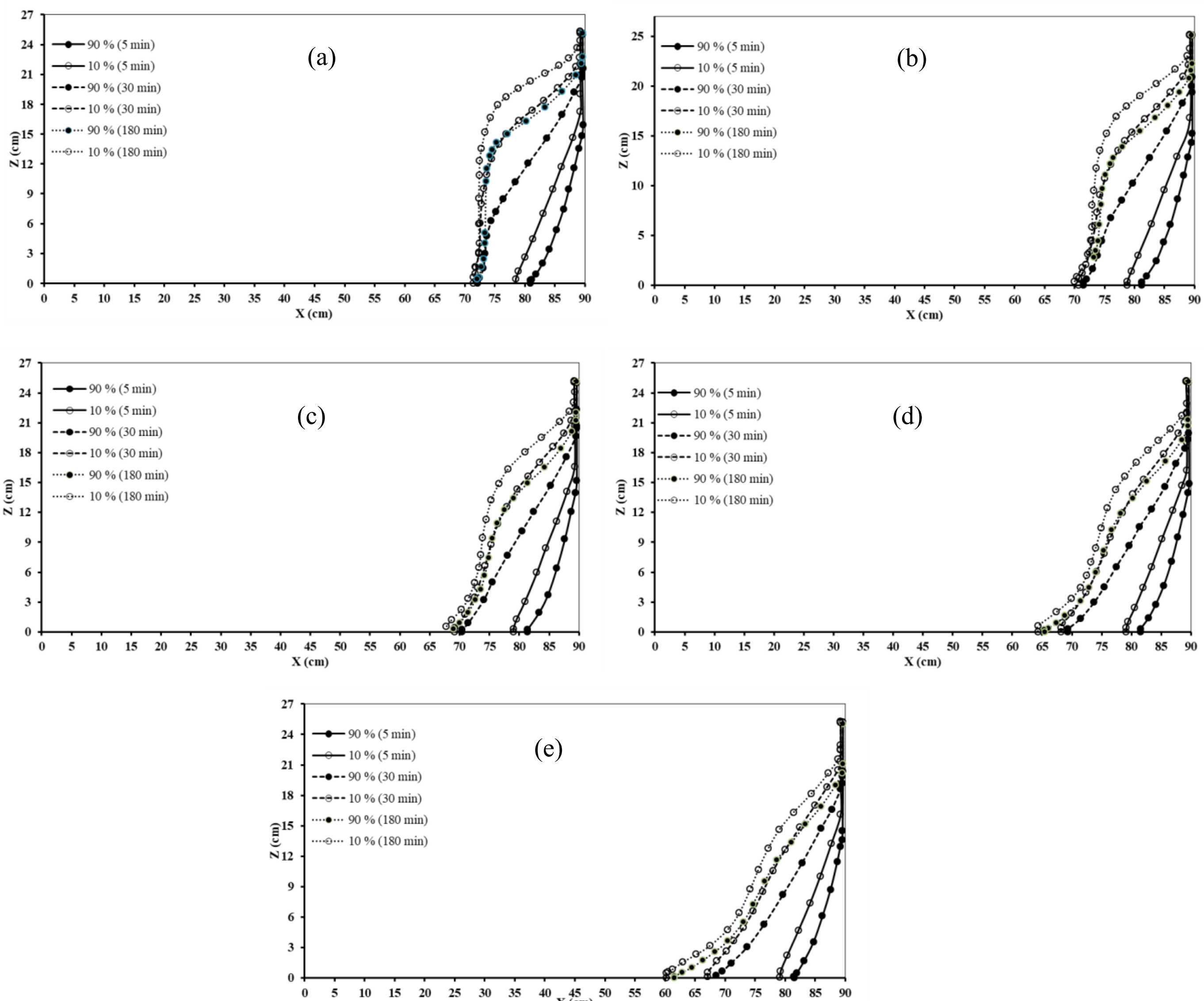

Fig.A7: Hydrodynamic behaviour of the seawater-freshwater mixing zone for a beach inclination angle equal to 0.0° for different bottom spacings of the cutoff wall S values: (a) 10, (b) 8, (c) 6, (d) 4 and (e) 2 cm.

Hydrodynamic behaviour of the seawater–freshwater mixing zone: influence of the bottom opening size below the cutoff wall in a 15° sloping coastal aquifer:

The seawater–freshwater mixing zone's temporal development across varying bottom opening sizes (S) beneath the cutoff wall in a coastal aquifer with a 15° slope is depicted in Fig. A8. The seawater front is moving slowly onshore, and the mixing zone is still somewhat small at the maximum bottom opening size (10 cm, Fig. A8a). Although the greater opening size offers less resistance to SWI, the aquifer's 15° slope still restricts the mixing zone's lateral spread. Because the smaller openings enable more efficient freshwater-saltwater interaction below the cutoff wall, the mixing zone widens as the bottom opening size lowers (8 cm and 6 cm in Figs. A8b and A8c), suggesting a faster inland migration of seawater. Consequently, the mixing zone expands more quickly. The seawater front penetrates farther inland, and the mixing zone is largest at the lowest bottom opening size (2 cm, Fig. A8e). A larger and more expansive mixing zone is made possible by the substantial decrease in SWI rate caused by the decrease in bottom opening size. This quick movement of the seawater is made possible by the higher gradient created by the 15° slope, which results in a sizable mixing zone at the end time steps. The impact of the inclination angle is evident when contrasting the outcomes with those in Fig. A7, which depicts a coastal aquifer with a 0° slope (vertical beach). Because the flow dynamics in Figure 8 are less affected by gravity than in the sloped case, the mixing zone expands more slowly and is more limited in the horizontal direction. For similar bottom opening sizes, the mixing zone in the 0° slope arrangement has a smaller area and increases more slowly over time, suggesting that the lack of a slope lessens the seawater's horizontal spreading. On the other hand, the 15° sloping aquifer in Fig.A8 permits more seawater to disperse laterally, especially when the size of the bottom aperture shrinks. Because there is a greater hydraulic gradient in the slope's direction, the mixing zone area is always greater than it would be at 0°. Furthermore, the narrower bottom holes produce a more noticeable SWI, which is consistent with the pattern in Fig. A7. Nonetheless, the degree of inclination speeds up the mixing zone's expansion both horizontally and vertically, highlighting the joint impact of aquifer slope and bottom opening size on the dynamics of the seawater–freshwater interface.

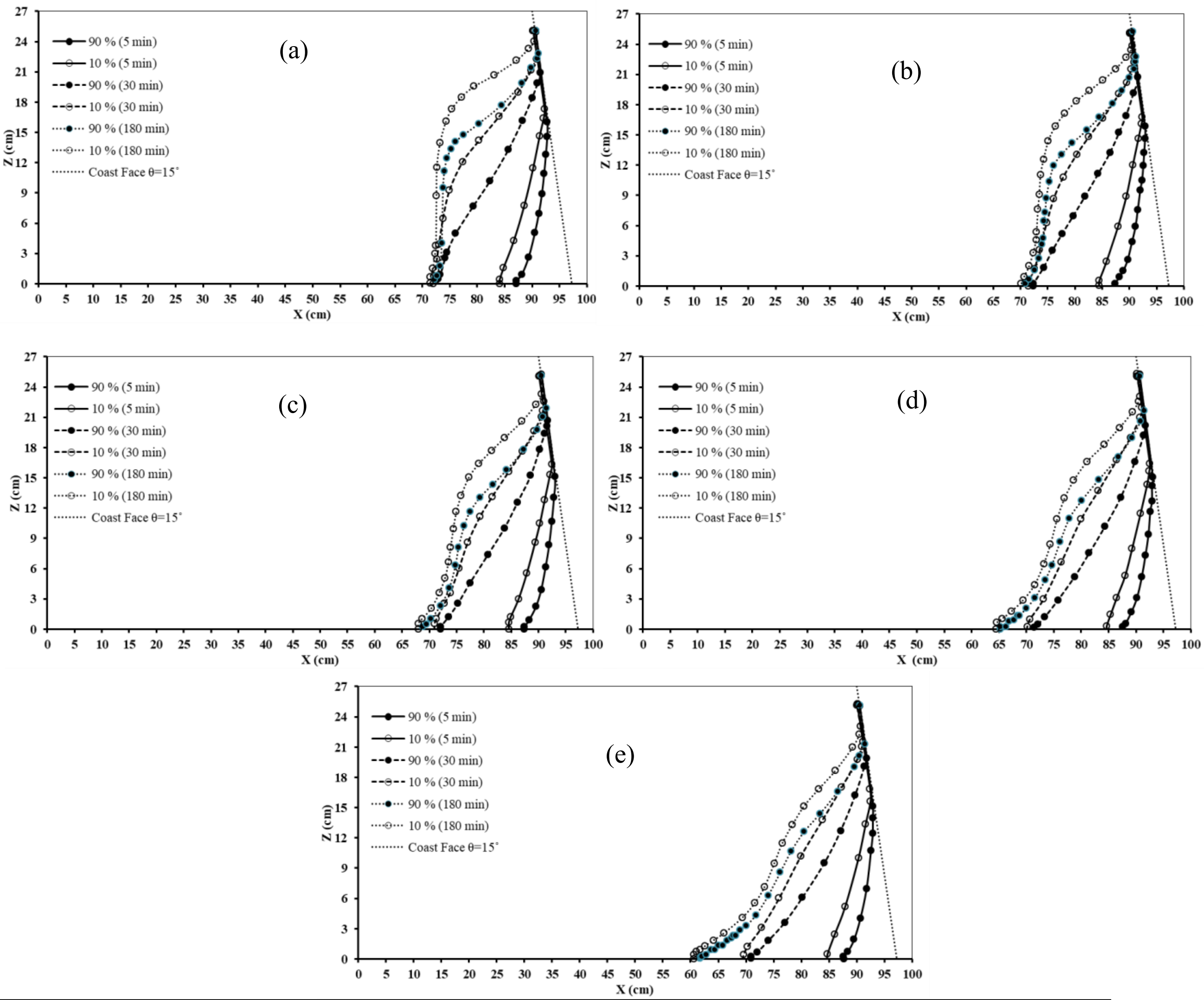

Fig.A8: Hydrodynamic behaviour of the seawater-freshwater mixing zone for a beach inclination angle equal to 15º, for different bottom opening sizes of the cutoff wall S values: (a) 10, (b) 8, (c) 6, (d) 4, and (e) 2 cm.

Hydrodynamic behaviour of seawater–freshwater mixing zone: influence of bottom opening size below the cutoff wall in a 30° sloping coastal aquifer:

The seawater–freshwater mixing zone's temporal evolution under different bottom opening sizes (S) beneath the cutoff wall in a coastal aquifer with a 30° slope is shown in Fig. A9. The seawater front moves slowly inland, and the mixing zone is comparatively limited at the maximum bottom opening size (10 cm, Fig. A9a). The mixing zone gets wider as the bottom opening size gets smaller (8 cm, 6 cm, 4 cm, and 2 cm in Figs. A9b to A9e), which suggests that the seawater is moving inland more quickly. The greater invasion of saltwater into the freshwater domain is made possible by the more effective interaction between the freshwater and saltwater due to the smaller bottom opening size. The mixing zone is greatest at the narrowest bottom opening size (2 cm, Fig. A9e), with the seawater front spreading well inland. The effect of the slope is clearly visible when contrasted to the vertical beach coastal aquifer (0° slope) in Fig. A7. The mixing zone in Fig. A9 stays comparatively limited in the horizontal plane and expands more slowly over time. Consequently, for the same bottom opening sizes, the mixing zone area is reduced. On the other hand, the higher hydraulic gradients in Fig. A8 and Fig. A9, which depict sloping coastal aquifers with 15° and 30° inclinations, improve the SWI. Seawater moves inland more quickly and widely because of the increased inclination degrees in Fig. 7 (15°) and Fig. 8 (30°). The mixing zone in the 30° sloping aquifer is noticeably larger than the zones seen in the 0° and 15° slope configurations, which is especially noticeable at the narrowest bottom opening sizes (2 cm). The mixing zone expands more quickly, particularly horizontally, when a steeper gradient and a smaller bottom opening size are combined. The mixing zone width increases most noticeably at the 2 cm bottom opening size (Fig. A9e) in the 30° inclined aquifer (Fig. A9). This implies that, in contrast to the 15° sloping system in Fig. A8, the steeper aquifer slope promotes more rapid and extensive SWI. The saltwater's lateral flow is amplified by the higher hydraulic gradient caused by the increased inclination angle, creating a mixing zone that is larger and grows faster. Furthermore, the analysis of Fig. A7 and Fig. A8 reveals that the mixing zone is wider at a 30° inclination than it is at a 15° one, suggesting that the coastal aquifer's angle plays a crucial role in defining the size and dynamics of the freshwater–saltwater mixing zone. Slope plays an important role in regulating SWI and mixing zone growth, as evidenced by the faster lateral dispersion at the 30° slope.

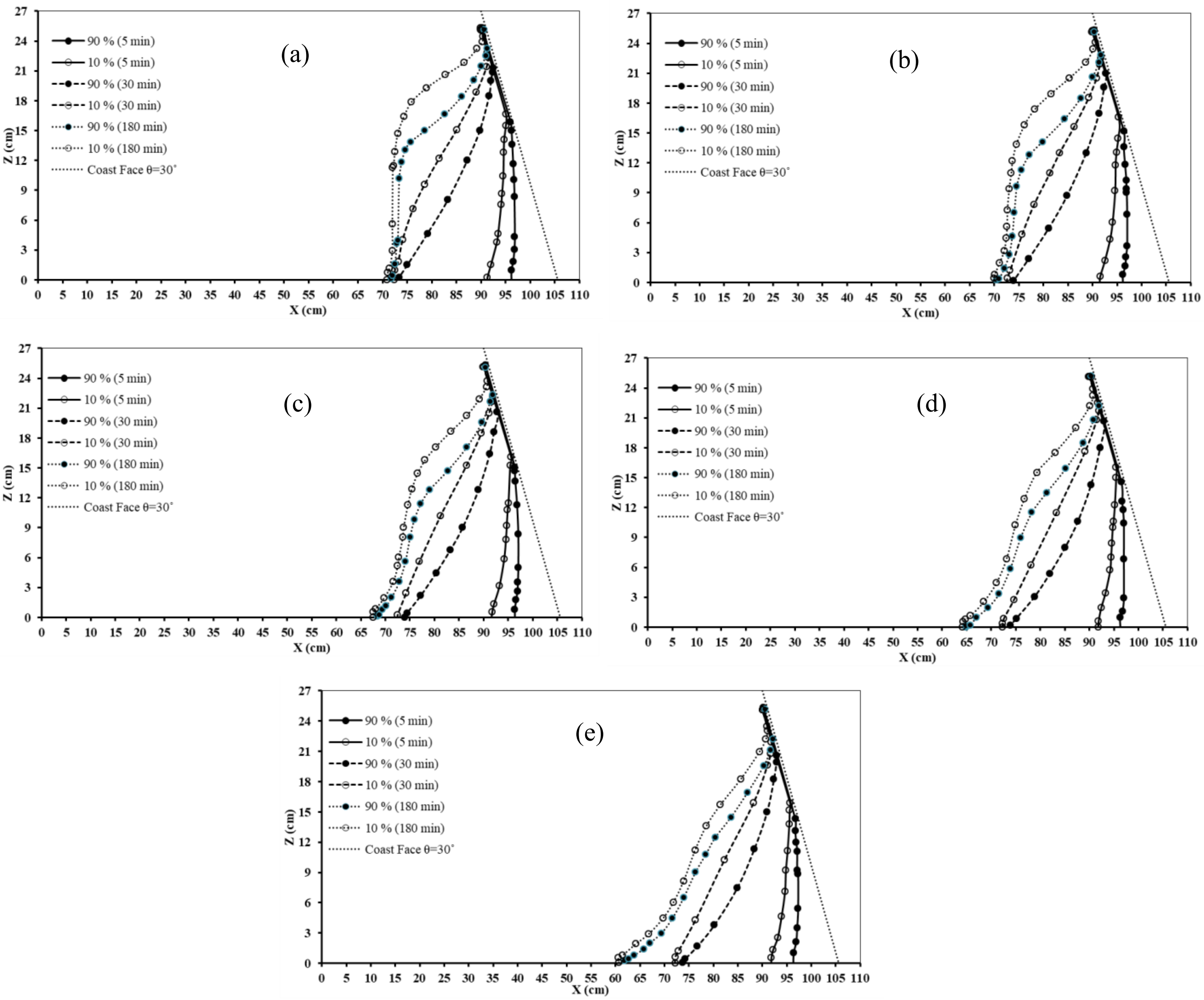

Fig.A9: Hydrodynamic behaviour of the seawater-freshwater mixing zone for a beach inclination angle equal to 30º, for different bottom opening sizes of the cutoff wall S values: (a) 10, (b) 8, (c) 6, (d) 4, and (e) 2 cm.

Hydrodynamic behaviour of the seawater–freshwater mixing zone: influence of bottom opening size below the cutoff wall in a 45° sloping coastal aquifer

The time evolution of the seawater–freshwater mixing zone in a 45° inclined coastal aquifer across various bottom opening sizes (S) beneath a cutoff wall is shown in Fig. A10. The seawater front moves slowly onshore, and the mixing zone is still somewhat small at the maximum bottom opening size (10 cm, Fig. A10a). The width of the mixing zone is constrained by the comparatively greater opening size, which permits restricted saltwater input. The mixing zone gets wider as the bottom opening size gets smaller (8 cm, 6 cm, 4 cm, and 2 cm in Figs. A10b–A10e), which suggests that the seawater is moving inland more quickly.

The mixing zone is the greatest when the bottom opening size is the smallest (2 cm, Fig. A10e), and the saltwater front extends far inland, exhibiting the fastest SWI. By comparing the outcomes of Fig. 10 (45° slope) with the vertical beach (0° slope) aquifer in Fig. A7, the 15° sloped beach aquifer in Fig. A8 and the 30° sloped beach aquifer in Fig. A9, it is evident how the coastal aquifer slope affects the seawater–freshwater mixing zone. In Fig. A7, the vertical beach aquifer's mixing zone remains more limited horizontally and expands more slowly than that of the sloped aquifers. A narrower and slower-expanding mixing zone results from the lack of a slope, which restricts the saltwater's lateral dispersion. In contrast, the sloping layouts improve SWI due to the higher hydraulic gradient. Because of the greater gradients in Figs. A8 (15° slope) and A9 (30° slope), the seawater front moves onshore more swiftly. Comparing these figures to Fig. A7, the mixing zones get larger, especially at lower bottom opening sizes where the faster SWI results in wider mixing zones. Seawater penetrates the 30° sloping aquifer (Fig. A9) quickly in both horizontal and vertical directions, resulting in the most noticeable increase in the mixing zone area. Fig. 10 (45° sloped aquifer) shows the most extensive mixing zone, particularly at the smallest bottom opening size (2 cm, Fig. 10e). The combination of the larger slope and smaller opening size facilitates the most rapid and widespread SWI, resulting in the largest mixing zone at each time step. The 45° slope produces the fastest inland movement of saltwater, leading to an even more pronounced lateral spread of the mixing zone compared to the 30° sloped aquifer in Fig. A10. The hydraulic gradient in the 45° aquifer is steeper, accelerating the saltwater front's movement, especially in the horizontal direction.

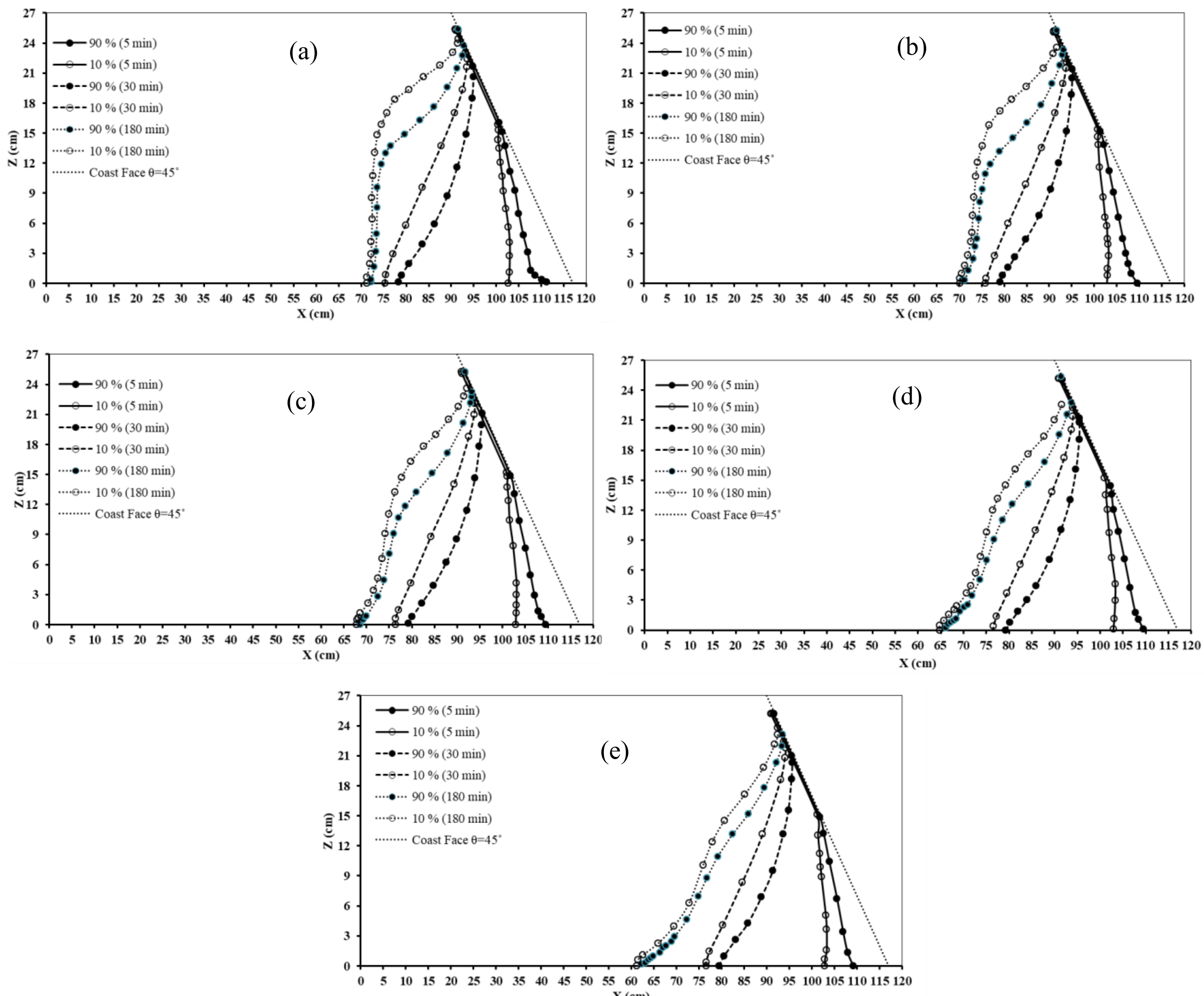

Fig. A10: Hydrodynamic behaviour of the seawater-freshwater mixing zone for a beach inclination angle to equal 45°, for different bottom opening sizes of the cutoff wall S values: (a) 10, (b) 8, (c) 6, (d) 4, and (e) 2 cm.

Cutoff walls had no effect on the invasion velocity and the mixing zone structure at 30 minutes. This is because the blocking impact had not yet occurred due to the saltwater wedge toe remaining away from the cutoff wall. The width and shape of the mixing zone stayed mostly unchanged, and the seawater front proceeded inland at a speed comparable to the control scenario (SWI without cutoff wall). The seawater wedge faced significant hydrodynamic resistance as it got closer to the cutoff wall, particularly because of the diverging freshwater flow through the bottom opening. This increased vertical mixing and created a wider mixing zone by upsetting the flow fields both inland and seaward of the wall. The combination of the salt wedge's upward displacement and the backflow and circulation of saltwater close to the bottom opening caused the mixing zone to greatly increase in both width and height, particularly in the average saturated aquifer depth. In cutoff wall scenarios, the average mixing zone width (MZW) was continuously greater, and the difference grew over time.

Larger openings permitted faster freshwater outflow, which accelerated the inland passage of the saltwater front once the toe reached the wall, according to simulations using a range of bottom opening sizes (2–10 cm). Smaller openings also produced the broadest mixing zones at a steady state and a more noticeable upward movement of the mixing zone. Differences in the mixing zone width, subsurface groundwater discharge (SGD) and toe length (T_L) were minimal in the beginning. Variations in these factors made it easy to discern between various aperture widths once the saltwater toe had reached the wall. In smaller-opening conditions, the SGD dropped further, increasing saltwater accumulation and residence time. Furthermore, as the bottom opening size increased, the central mixing zone width (W_2) decreased, emphasising the wall's hydraulic constraint regulating function. Though their impact is time-dependent and only becomes noticeable once the saltwater toe reaches the wall, cutoff walls, especially those with narrow bottom openings, are useful in controlling seawater intrusion and enlarging the mixing zone.

Impact of longitudinal despersivity and transverse despersivity on seawater-freshwater mixing zone:

Fig. A11 illustrates the simulated evolution of the saltwater-freshwater mixing zone under a condition of increased longitudinal dispersivity equal to $5\alpha_l$, displayed for three selected time intervals (5, 30, and 180 minutes) across various four sloping beach configurations (a-d). At the early stage of the simulation (5 minutes; a1-d1), the seawater intrusion wedge remains concentrated near the coastal boundary; however, compared with lower dispersivity scenarios, the interface between seawater and freshwater is already noticeably broadened. The seawater-freshwater mixing zone exhibits a thicker transition zone, representing enhanced spreading along the primary groundwater flow direction. The seawater intrusion wedge begins to lose its sharpness, and a smoother concentration gradient forms between the seawater and freshwater fluids.

By 30 minutes (a2–d2), the influence of the higher longitudinal dispersivity values develops more pronounced. The seawater intrusion advances further inland whereas simultaneously elongate horizontally. The interface between seawater and freshwater expands significantly, and the concentration contours become more gradual and widely spaced. In cases with inclined or sloped boundaries (c2 and d2), the seawater intrusion wedge adapts to the geometry, spreading preferentially along the slope boundary and forming a more extended, fan-shaped transition region. The enhanced dispersive impact promotes stronger interaction between the seawater and the freshwater, increasing the width of the seawater-freshwater mixing zone. At the later time of the simulation (180 minutes; a3–d3), the system approaches a more developed seawater intrusion pattern. The seawater intrusion wedge penetrates deeper inland, and the seawater-freshwater mixing zone becomes substantially thicker than in earlier stages. The seawater-freshwater interface is no longer sharply defined; instead, a broad, smoothly varying salinity gradient dominates the transition area. Higher values of longitudinal dispersivity intensifies axial spreading, resulting in greater dilution at the leading edge of the seawater intrusion wedge. In the sloped boundary tested configurations, the seawater intrusion wedge geometry becomes more curved and extended, emphasizing the role of domain shape in controlling dispersive seawater-freshwater mixing.

Overall, increasing the longitudinal dispersivity to $5\alpha_l$ amplifies the horizontal spreading of saline water, enlarges the seawater–freshwater mixing zone, reduces concentration gradients at the seawater-freshwater interface, and accelerates inland seawater intrusion across all modelled tested configurations.

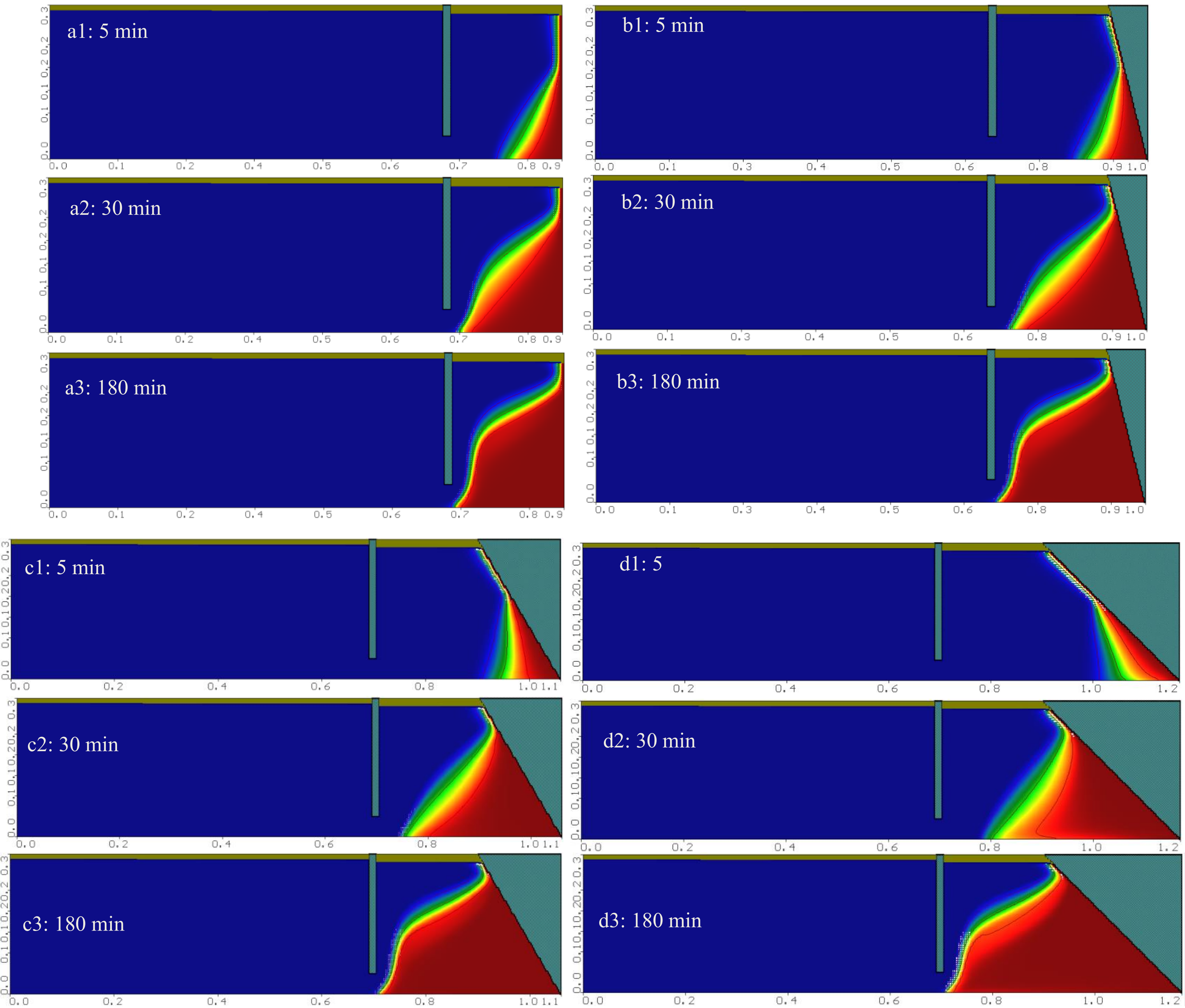

Fig. A11: Steady state seawater intrusion for sloped beach coastal aquifers for the case with below cutoff wall opening (S=4 cm), for increasing longitudinal despersivity ($5\alpha_L$) for various inclination angles of the beach face: (a) 0.0^o, (b) 15°, (c) 30° and (d) 45°; the black lines indicate the 10, 50 and 90% isohalines of the mixing zone.

Fig. A12 illustrates the development of the seawater-freshwater mixing zone when the transversal dispersivity is increased to $2\alpha_t$, displayed at 5, 30, and 180 minutes for four various sloping beach boundary configurations (a–d). At the initial stage of the simulation (5 minutes; a1–d1), the seawater intrusion wedge is still confined near the coastal seawater boundary. Though, compared with lower transverse dispersivity conditions, the seawater-freshwater interface between saline and freshwater appears slightly thicker in the vertical direction. The transition zone displays enhanced lateral smoothing perpendicular to the main groundwater flow direction, while the seawater inland penetration length remains limited. By 30 minutes (a2–d2), the impact of increased the value of transverse dispersivity becomes clearer. The seawater-freshwater mixing zone expands more prominently in the vertical direction, resulting in a thicker and more diffused seawater-freshwater interface. Instead of a narrow transition line, the saline intrusion wedge develops a wider brackish area. In the sloping seawater boundary cases (c2 and d2), dispersion perpendicular to flow causes the seawater-freshwater mixing layer to spread upward and downward along the inclined beach face, producing a smoother curvature of the seawater intrusion wedge. The inland advance of the seawater intrusion wedge toe is not dramatically extended, but the interface loses sharpness and becomes more gradational. At the later time of the simulation (180 minutes; a3–d3), the seawater-freshwater transition zone is substantially broadened. The higher value of the transversal dispersivity enhances cross-sectional seawater-freshwater mixing, leading to a thicker intermediate salinity zone throughout the seawater intrusion wedge. The seawater intrusion wedge maintains a similar horizontal reach compared to earlier stages; however, its internal structure becomes more diffuse. In geometries with inclined beach coastal boundaries (c3 and d3), the seawater intrusion wedge exhibits a more rounded and vertically expanded profile, reflecting intensified seawater-freshwater mixing across streamlines rather than along them. Overall, increasing the value of the transversal dispersivity to $2\alpha_t$ primarily amplifies the vertical spreading and interfacial thickening of the seawater-freshwater mixing zone. Unlike longitudinal dispersivity, which mainly controls inland elongation, higher value of transverse dispersivity broadens the transition region, smooths salinity gradients, and promotes stronger crossflow mixing without significantly increasing the horizontal seawater intrusion wedge length.

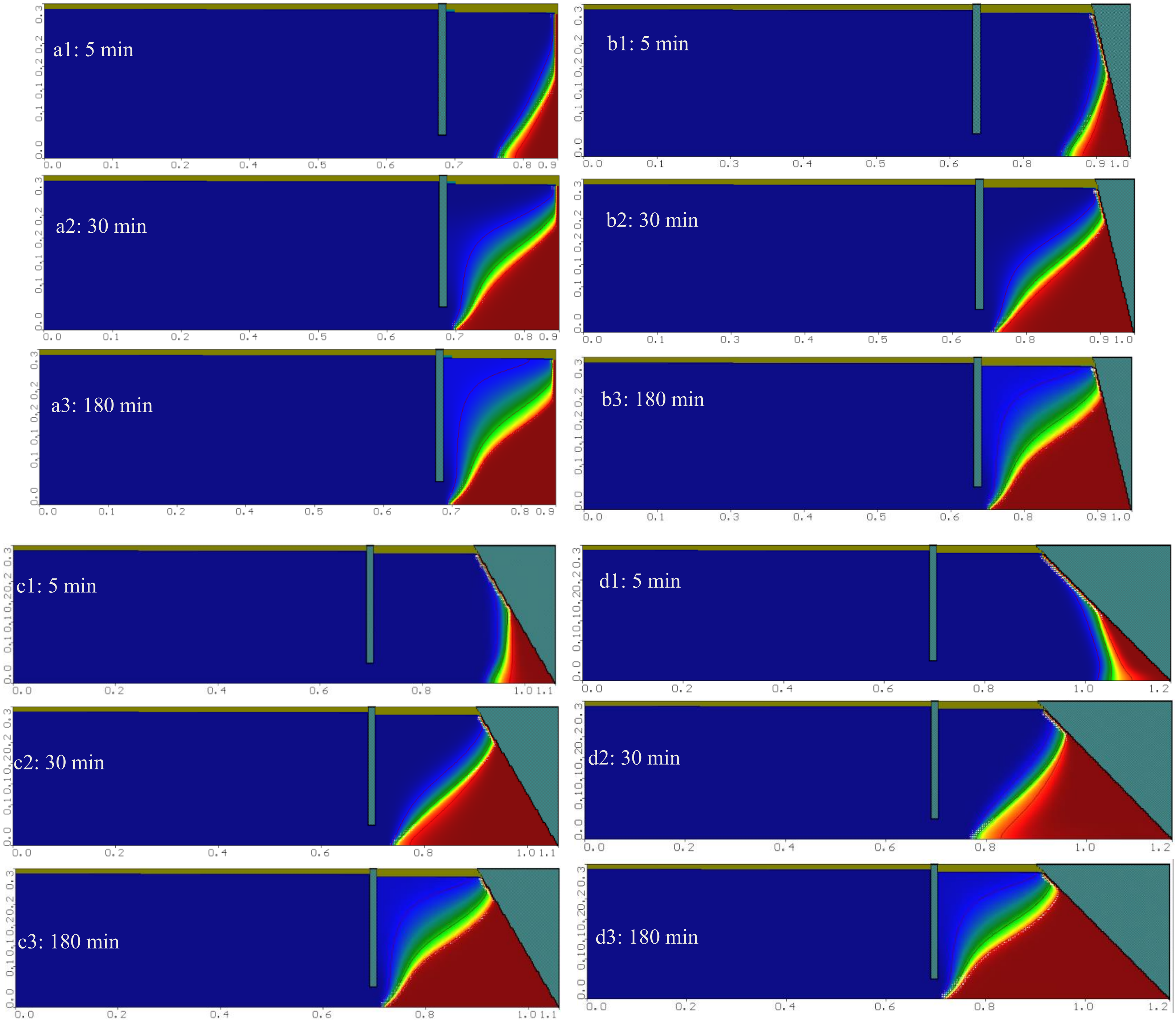

Fig. A12: Steady state seawater intrusion for sloped beach coastal aquifers for the case with below cutoff wall opening (S=4 cm), for increasing transverse despersivity ($2\alpha_T$) for various inclination angles of the beach face: (a) 0.0^o, (b) 15°, (c) 30° and (d) 45°; the black lines indicate the 10, 50 and 90% isohalines of the mixing zone.

Impact of hydraulic conductivity on seawater-freshwater mixing zone:

Fig. A13 illustrates the progress of the seawater-freshwater mixing zone when the hydraulic conductivity is minimized to 0.50K, presented at 5, 30, and 180 minutes for different four inclination angle boundary configurations (a–d). At the early stage of the simulation (5 minutes; a1–d1), the seawater intrusion is highly limited close to the coastal saltwater boundary. The reduced hydraulic conductivity limits the groundwater velocity, thereby reducing the inland movement of saltwater. The seawater intrusion wedge seems narrow and compressed, with a relatively steep concentration gradient at the seawater-freshwater interface. In the sloping beach boundary cases (c1 and d1), the seawater intrusion wedge remains confined close to the inclined beach face, viewing minimal inland seawater intrusion advancement. By 30 minutes (a2–d2), the impact of lower hydraulic conductivity develops more evident. Compared with the higher-conductivity scenarios, the inland migration of the seawater intrusion toe progresses more slowly. The seawater-freshwater mixing zone develops gradually, but its horizontal extension remains limited. The transition zone between seawater and freshwater thickens slightly because of dispersion; though, the overall seawater intrusion wedge length is shorter. In configurations with inclined beach face coastal boundaries (c2 and d2), the seawater intrusion interface follows the slope but keeps a compact geometry, indicating that reduced flow capacity suppresses rapid spreading. At 180 minutes at the end of the simulation (a3–d3), the system approaches a more stabilized intrusion pattern under constrained flow conditions. The seawater intrusion wedge extends further inland than at earlier times, yet its reach is prominently declined compared to the cases with higher value of hydraulic conductivity. The seawater-freshwater mixing zone remains relatively concentrated, with sharper salinity gradients and a more distinct separation between the seawater and freshwater. In sloped geometries (c3 and d3), the seawater intrusion wedge forms a curved profile along the boundary, but its seawater penetration length and thickness remain moderated by the reduced hydraulic conductivity. Overall, declining the value of hydraulic conductivity to 0.50K significantly slows the seawater intrusion, shortens the inland extent of the seawater intrusion wedge, and maintains a comparatively sharper seawater-freshwater mixing interface. The reduced hydraulic conductivity restricts the groundwater flow velocity, so limiting both the rate and magnitude of seawater-freshwater interaction across all tested modeled configurations.

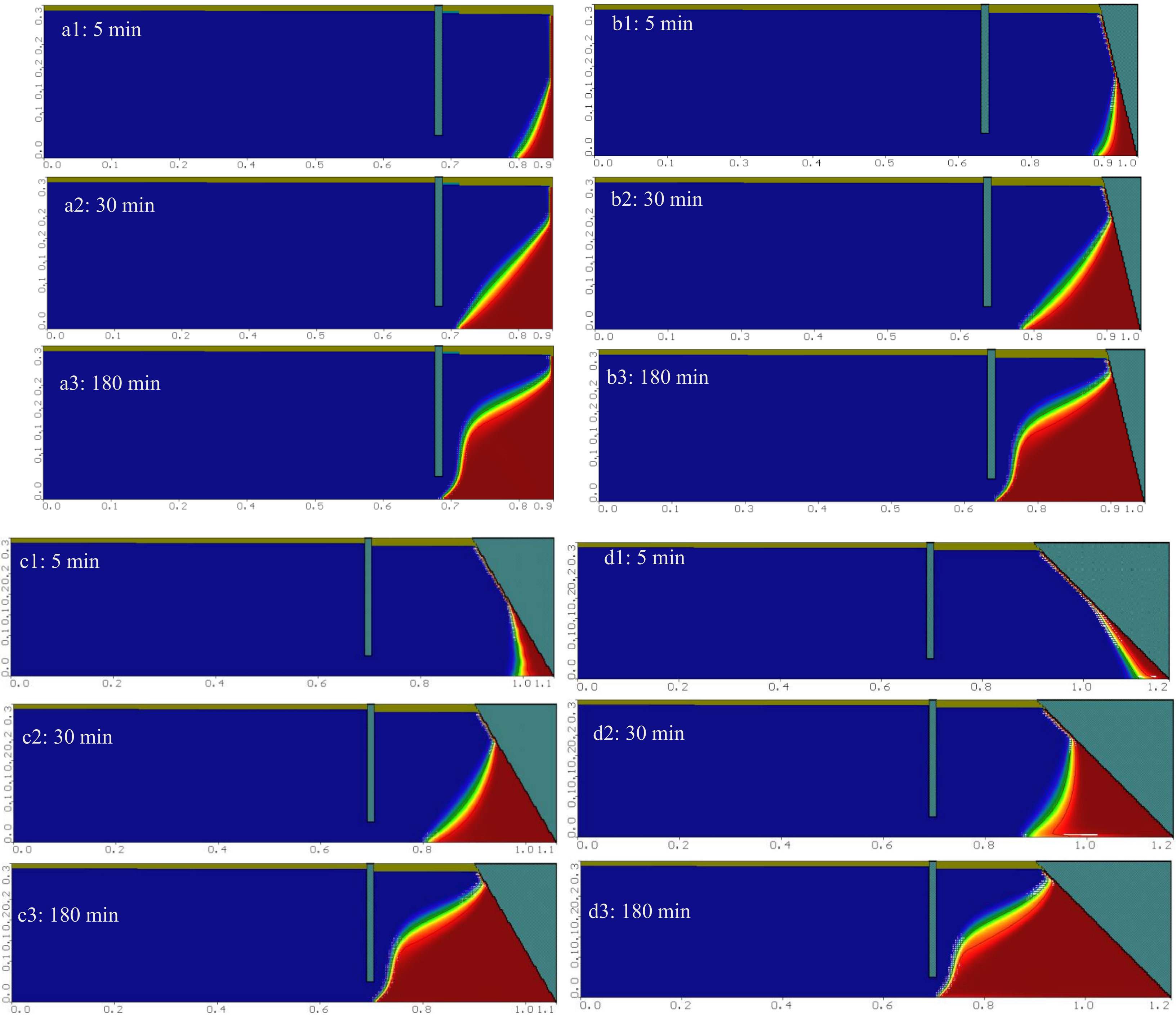

Fig. A13: Steady state seawater intrusion for sloped beach coastal aquifers for the case with below cutoff wall opening (S=4 cm), for minmizing hydraulic conductivity (0.5K) for various inclination angles of the beach face: (a) 0.0^o, (b) 15°, (c) 30° and (d) 45°; the black lines indicate the 10, 50 and 90% isohalines of the mixing zone.

Fig. A14 illustrates the temporal expansion of the seawater-freshwater mixing zone when the hydraulic conductivity is further declined to 0.25K, evaluated after 5, 30, and 180 minutes for the different four sloping beach boundary arrangements (a–d). The additional reduction in hydraulic conductivity imposes stronger resistance to groundwater movement, substantially reducing the rate of seawater intrusion advancement. At the initial stage of the simulation (5 minutes; a1–d1), seawater intrusion remains highly localized near the coastal beach boundary in all tested configurations. The seawater intrusion wedge is obviously thinner and more compressed than in the 0.50K case, demonstrating that the lower hydraulic conductivity substantially limits the interaction between seawater and freshwater. The seawater intrusion interface between the two fluids is steep and sharply defined, with only a narrow seawater-freshwater mixing width. In the tested inclined beach boundary models (c1 and d1), the seawater intrusion wedge closely follows the coastal slope while exhibiting very limited seawater intrusion inland penetration length, representing the strong control of reduced hydraulic conductivity on the early stage of intrusion.

After 30 minutes (a2–d2), the seawater intrusion wedge toe advances landward, but its progression remains restrained. Although the transition zone becomes more visible due to dispersive mixing, the overall seawater intrusion wedge length is still shorter than that observed for 0.50K. The freshwater domain continues to occupy most of the aquifer, although the seawater intrusion wedge toe remains concentrated near the coastal seawater boundary. In the tested sloping configurations (c2 and d2), the seawater-freshwater interface develops a smooth curved shape along the inclined boundary; however, both the horizontal extent and thickness of the intrusion zone remain comparatively limited. By 180 minutes (a3–d3), a more developed seawater intrusion pattern emerges, yet the seawater intrusion wedge remains distinctly compact. The toe of seawater intrusion wedge extends inland relative to the earlier times of the simulation, but its reach is still substantially restricted by the low value of hydraulic conductivity. The seawater-freshwater mixing region demonstrates gradual curvature and moderate thickening, though the transition between freshwater and saline water remains relatively sharp. The inclined-boundary cases continue to exhibit a curved interface aligned with the slope, but the saline wedge occupies a smaller portion of the aquifer than would be expected under higher-hydraulic conductivity conditions. Overall, reducing the value of hydraulic conductivity from 0.50K to 0.25K further reduces seawater intrusion by decreasing groundwater flow capacity and limiting salt transport. As a result, the seawater intrusion wedge remains more compact, advances more slowly, and occupies a smaller area of the aquifer across all tested sloping boundary configurations.

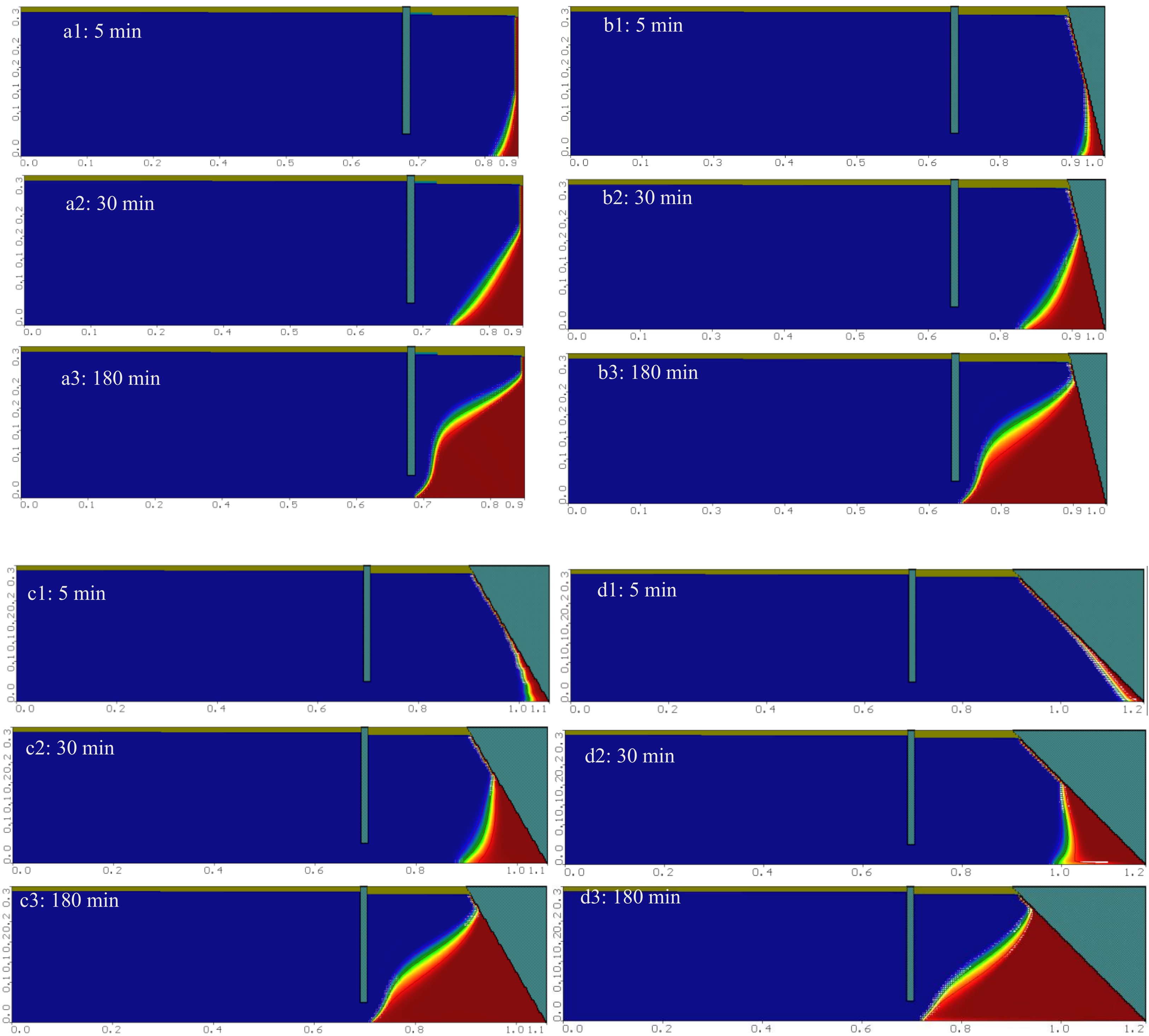

Fig. A14: Steady state seawater intrusion for sloped beach coastal aquifers for the case with below cutoff opening (S=4 cm), for minmizing hydraulic conductivity (0.25K) for various inclination angles of the beach face: (a) 0.0°, (b) 15°, (c) 30° and (d) 45°; the black lines indicate the 10, 50 and 90% isohalines of the mixing zone.

Impact of cutoff wall location on seawater-freshwater mixing zone:

Fig. A15 displays the temporal development of the seawater-freshwater mixing zone when the cutoff wall is located at 0.75Xc, assessed at 5, 30, and 180 minutes for the different four coastal beach boundary configurations (a–d). Moving the cutoff wall closer to the seawater boundary modifies the groundwater flow field and alters the pattern of seawater encroachment. The results prove that the shortened wall distance influences both the shape and extent of the seawater intrusion wedge while maintaining the cutoff wall's role as a hydraulic barrier. At the early stage of the simulation (5 minutes; a1–d1), saltwater remains concentrated close to the coastal beach boundary, forming a narrow seawater intrusion wedge with a well-defined interface. The cutoff wall restricts inland movement of seawater, causing the intrusion wedge toe to develop primarily seaward of the barrier. In the vertical-boundary configurations (a1), the seawater-freshwater mixing zone is confined to a limited region adjacent to the coast. For the sloping geometries (b1, c1 and d1), the seawater intrusion follows the inclined beach boundary and exhibits a slightly broader distribution near the seawater boundary, although inland penetration remains minimal. By 30 minutes (a2–d2), the seawater intrusion wedge expands, and the transition zone becomes more pronounced. The seawater intrusion wedge toe advances landward beneath the cutoff wall, producing a curved interface that reflects the interaction between density-driven flow and the hydraulic obstruction. The seawater-freshwater mixing zone thickens as dispersion develops, but the cutoff wall continues to limit rapid inland seawater migration. In configurations b2, c2 and d2, the seawater intrusion wedge aligns with the sloping coastal face and extends upward along the incline, creating a broader transition region than in the vertical-boundary cases.

At 180 minutes (a3–d3), the seawater intrusion system approaches steady state condition characterized by a stable wedge geometry. The seawater intrusion occupies a larger portion of the coastal aquifer than at earlier times, while the freshwater zone remains dominant inland of the cutoff wall. The seawater-freshwater interface exhibits a smooth, landward-curving profile, indicating sustained mixing near the seawater intrusion wedge. In the tested inclined-boundary models (b3, c3 and d3), the seawater intrusion wedge becomes more elongated and follows the coastal slope, resulting in a wider seawater-freshwater mixing region and a more gradual salinity transition compared with the vertical-boundary configurations. Overall, positioning the cutoff wall at 0.75Xc allows seawater intrusion to develop while still providing substantial protection to the inland aquifer. The cutoff wall limits the advance of the saline intrusion wedge and promotes the formation of a concentrated transition zone near the coastal boundary. Across all tested configurations, the seawater-freshwater interface evolves from a narrow coastal intrusion at early times to a broader and more stable mixing zone at later stages, with the greatest spreading occurring in the sloping-boundary cases due to geometric effects on flow and dispersion.

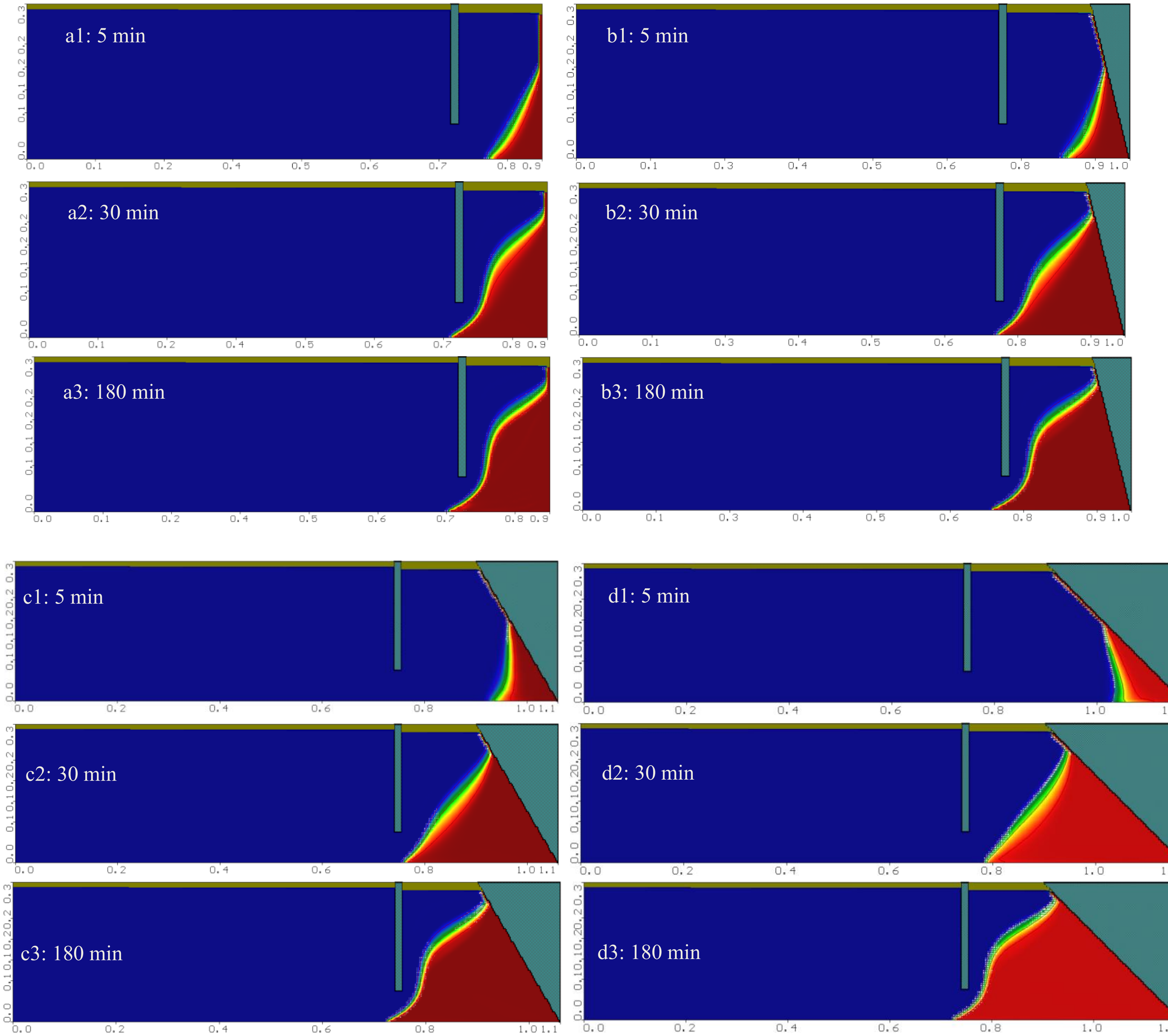

Fig. A15: Steady state seawater intrusion for sloped beach coastal aquifers for the case with below cutoff wall opening (S=6 cm), for minmizing cutoff wall distance ($0.75X_c$) for various inclination angles of the beach face: (a) 0.0°, (b) 15°, (c) 30° and (d) 45°; the black lines indicate the 10, 50 and 90% isohalines of the mixing zone.

Fig. A16 demonstrates the development of the seawater-freshwater mixing zone when the cutoff wall is repositioned to $0.5X_c$, corresponding to a position closer to the shoreline than the previously tested configuration. The seawater distributions are displayed at 5, 30, and 180 minutes for the different four boundary geometries (a–d). The reduced cutoff wall distance modifies the flow field near the coast boundary, influencing both the shape of the seawater intrusion wedge and the extent of freshwater protection within the aquifer. At the initial stage of the simulation (5 minutes; a1–d1), seawater intrusion remains concentrated in a narrow region adjacent to the coastal beach boundary. The proximity of the cutoff wall limits the available flow space between the shoreline boundary and the barrier, causing the seawater intrusion wedge toe to develop within a confined zone. The seawater intrusion wedge exhibits a steep interface and a relatively thin seawater-freshwater mixing zone. In the sloping-boundary configurations (b1, c1 and d1), the mixing zone follows the inclined coastal face while remaining largely restricted to the seaward side of the cutoff wall. By 30 minutes (a2–d2), the saline intrusion wedge becomes more developed and the transition zone between seawater and freshwater broadens due to dispersive mixing. The seawater-freshwater interface curves upward toward the coastal boundary while extending beneath the cutoff wall. Despite this growth, the intrusion remains concentrated within the coastal portion of the domain. In the tested inclined geometries (b2, c2 and d2), the seawater intrusion wedge expands along the sloping boundary and forms a wider transition zone than in the vertical-boundary cases, reflecting enhanced interaction between density-driven flow and the coastal boundary slope.

At 180 minutes (a3–d3), the seawater intrusion pattern reaches a more stable configuration. The seawater wedge penetrates farther inland than during earlier stages, but its evolution remains controlled by the hydraulic barrier. The seawater-freshwater mixing zone displays a smooth, curved profile with a clearly defined transition between seawater and freshwater. Configurations b3, c3 and d3 exhibit the most pronounced seawater intrusion wedge development, where the intrusion wedge extends upward along the inclined boundary and occupies a larger coastal area. Nevertheless, a substantial freshwater region persists inland of the cutoff wall, indicating continued effectiveness of the barrier. Reducing the cutoff wall distance from $0.75X_c$ to $0.5X_c$ enhances the barrier's ability to control seawater intrusion. The closer wall location confines the seawater intrusion wedge to the coastal zone, decreases the spatial extent of the mixing zone, and preserves a larger freshwater reserve inland. While seawater intrusion continues to evolve over time in all tested configurations, the $0.5X_c$ arrangement provides more effective suppression of seawater intrusion and yields a more compact and stable seawater-freshwater interface than the $0.75X_c$ case.

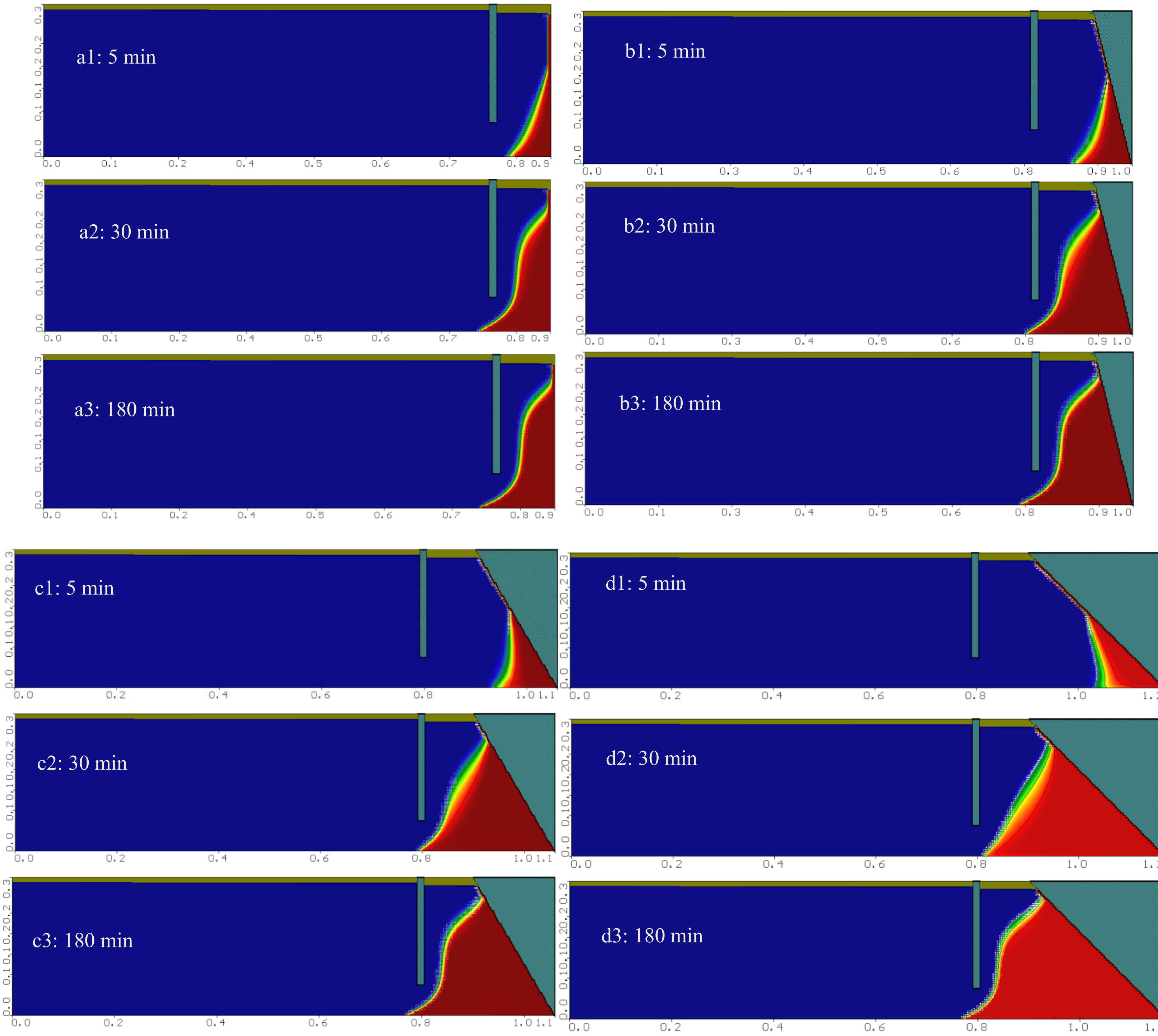

Fig. A16: Steady state seawater intrusion for sloped beach coastal aquifers for the case with below cutoff wall opening (S=6 cm), for minmizing cutoff wall distance (0.50X_c) for various inclination angles of the beach face: (a) 0.0^o, (b) 15°, (c) 30° and (d) 45°; the black lines indicate the 10, 50 and 90% isohalines of the mixing zone.

Fig. A17 illustrates the temporal evolution of the seawater-freshwater mixing zone when the cutoff wall is positioned at 0.25Xc, representing the closest wall location to the coastal line among the investigated cases. Seawater intrusion distributions are presented at 5, 30, and 180 min for the various four coastal boundary configurations (a–d). Positioning the cutoff wall closer to the coastal boundary significantly alters the flow regime by intercepting seawater intrusion at an earlier stage, thereby restricting the growth of the seawater intrusion wedge and reducing the extent of the mixing zone. At the initial stage (5 min; a1–d1), seawater intrusion is confined to a very narrow zone immediately adjacent to the shoreline boundary. The seawater intrusion wedge remains highly localized between the coastal boundary and the cutoff wall, producing a steep and well-defined salinity interface. In comparison with the 0.5Xc and 0.75Xc tested cases, the seawater intrusion wedge occupies a prominently smaller area, representing that the reduced cutoff wall distance efficiently limits early-stage of seawater intrusion. In the sloping-boundary configurations (b1, c1 and d1), the seawater intrusion follows the inclined coastal face, but its inland expansion remains strongly constrained by the nearby barrier.

By 30 min (a2–d2), the seawater intrusion wedge becomes more developed, and the transition zone broadens slightly due to dispersion. Though, the seawater intrusion wedge remains concentrated within the narrow coastal compartment formed by the shoreline and the cutoff wall. The seawater-freshwater interface exhibits a pronounced upward curvature near the barrier location, reflecting the obstruction of density-driven inland flow. Although configurations b2, c2 and d2 show greater vertical spreading along the sloping coastal boundary, the overall extent of the mixing zone remains considerably smaller than that observed in the 0.5Xc and 0.75Xc configurations. At 180 min (a3–d3), the seawater intrusion reached steady state condition. The seawater intrusion wedge stabilizes within the coastal region and exhibits only limited inland penetration beyond the cutoff wall location. The mixing zone remains compact, with a relatively sharp freshwater-seawater transition. As in the previous cases tested, the inclined-boundary models (b3, c3 and d3) display slightly larger saline intrusion wedges and wider transition zones than the vertical-boundary model (a3), but the impact of coastal geometry is significantly reduced because the wall intercepts the seawater intrusion close to the shoreline.

Among the three wall locations, 0.25Xc provides the highest level of protection against seawater intrusion. Positioning the cutoff wall very close to the shoreline minimizes the available space for saline wedge development, resulting in the smallest mixing zone, the sharpest salinity transition, and the greatest preservation of inland freshwater resources. In comparison, the 0.5Xc configuration offers intermediate protection, while the 0.75Xc arrangement permits greater seawater intrusion wedge expansion and a broader seawater-freshwater mixing zone before the seawater intrusion encounters the hydraulic barrier. Consequently, reducing the cutoff wall distance progressively improves the effectiveness of seawater intrusion control and enhances aquifer freshwater security.

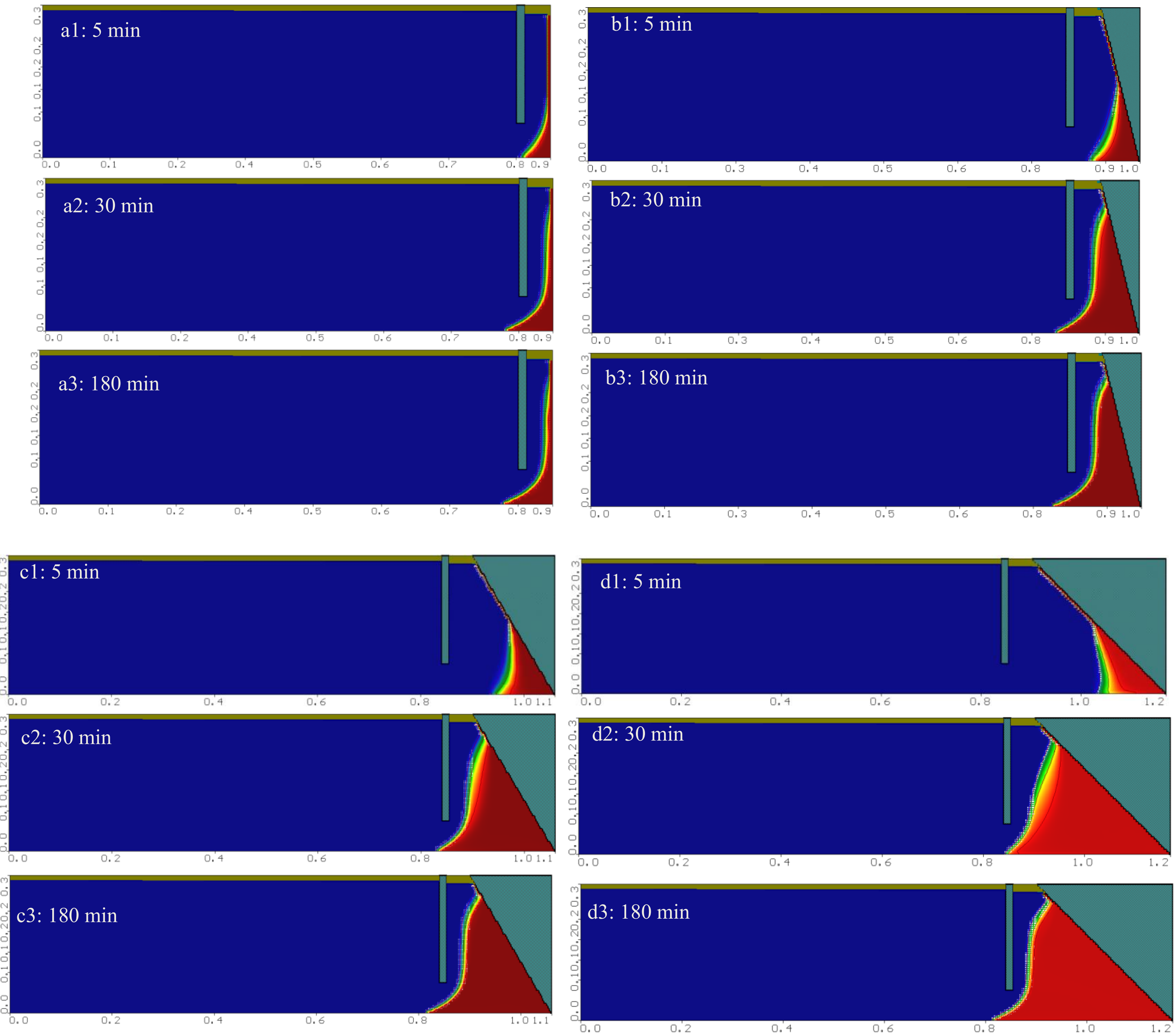

Fig. A17: Steady state seawater intrusion for sloped beach coastal aquifers for the case with below cutoff wall opening (S=6 cm), for minmizing cutoff wall distance (0.25X_c) for various inclination angles of the beach face: (a) 0.0°, (b) 15°, (c) 30° and (d) 45°; the black lines indicate the 10, 50 and 90% isohalines of the mixing zone.

The findings offer a thorough examination of the mixing zone and the hydrodynamic behaviour of SWI in coastal aquifers with cutoff walls. The figures show how the dynamics of the seawater-freshwater mixing zone are affected by several parameters, including the inclination angle of the aquifer (θ) and the bottom opening size of the cutoff wall (S). Across all figures, a consistent pattern shows that SWI rises quickly at first before slowing down and stabilising. The key variables, including subsurface groundwater discharge (SGD), seawater wedge length (T_L), mixing zone area (A_m), bottom width of the mixing zone (W_1), and average mixing zone width (MZW), gradually stabilise, reflecting this time-dependent behaviour. As the saltwater wedge gets closer to the cutoff wall, its increasing effect is thought to be the reason for the change from fast intrusion to stabilisation. The width of the seawater wedge and its invasion rate both rise rapidly in the early phases of the simulation, as seen with SGD and T_L, but the growth rate decreases as the system stabilises. This supports the findings of Chang et al. [44], which showed that when the seawater wedge approaches the barrier, the effect of physical barriers such as cutoff walls increases. According to these time-dependent dynamics, cutoff walls initially have little impact on SWI, but as the saltwater front gets closer to the physical barrier, their significance increases.

Controlling the pace of seawater invasion and the mixing zone's expansion depends critically on the size of the cutoff wall's (S) bottom opening. The results show that the seawater wedge length stabilises more quickly and the SGD decreases more quickly with smaller bottom openings (e.g., S = 2 cm, 4 cm). These findings are consistent with Chang et al. [44], who found that smaller cutoff apertures led to a faster reaction in lowering SWI and limiting the mixing zone's expansion. The parameters of the mixing zone, especially the area (A_M) and width (MZW), decreased more quickly in the early phases of the tested simulation due to the stronger barrier effect. Larger bottom apertures (S = 6 cm, 8 cm and 10 cm), on the other hand, permit more seawater to enter before the barrier's effects are recognised. As a result, the SGD reduces more slowly, and the seawater wedge and mixing zone stabilise more gradually. These results imply that the size of the bottom hole has an inverse relationship with the cutoff wall's ability to control SWI, with smaller openings offering superior intrusion resistance.

The coastal aquifer's inclination angle (θ) has a major impact on the mixing zone width and SWI dynamics. The results show that slower rates of seawater wedge growth and mixing zone extension are associated with steeper aquifers (higher θ). This is because the advancement of the seawater wedge is delayed in sloped aquifers due to the more complex hydraulic gradients. The SGD and seawater wedge length (T_L) results confirm that aquifers with a 0° slope (vertical beach aquifer) had the quickest rate of expansion in both dimensions. Both parameters increase more slowly as the inclination rises to 45°, which is indicative of the more intricate flow dynamics at higher angles. Similarly, for aquifers with higher inclination angles, the average mixing zone width (MZW) and the bottom width of the mixing zone (W_1) rise more slowly. This implies that steeper aquifers, maybe because of increasing vertical gradients and less lateral flow, impede the mixing zone's quick expansion. The rate of SWI and the subsequent formation of the mixing zone are significantly influenced by the aquifer's inclination angle. Because their flow patterns are more complicated, steeper aquifers respond to SWI more slowly. Ultimately, the inclination angle (θ) was found to be a crucial element in regulating the mixing zone and SWI dynamics. Steeper aquifers (θ = 45°) displayed slower rates of expansion, whereas vertical beach border aquifers (θ = 0°) displayed higher rates of SWI. Because sloped aquifers have different hydraulic gradients and flow dynamics, the rate of SWI is slower.

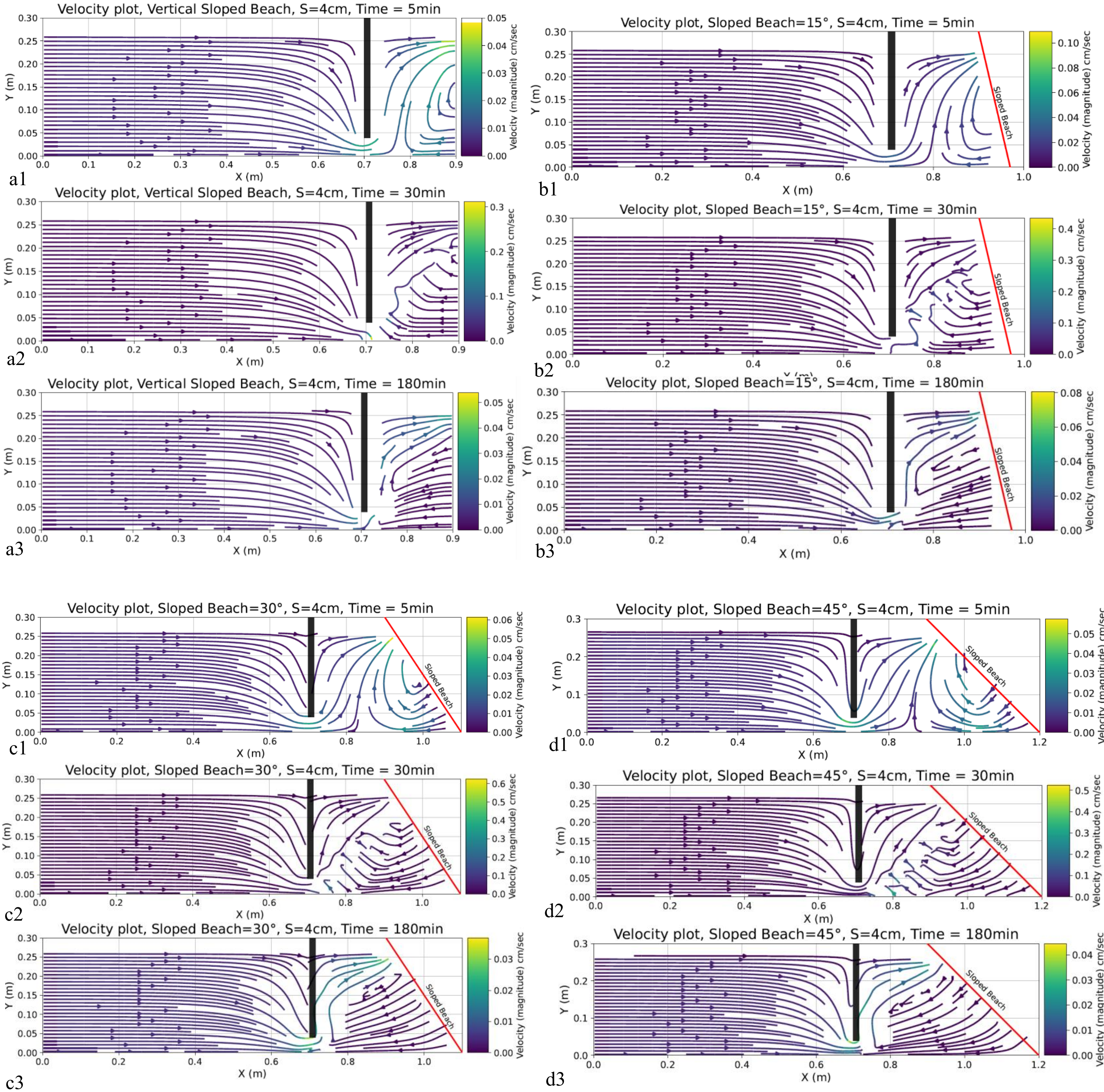

Fig. A18: Velocity plot for various sloped beach coastal aquifers: (a) vertical, (b) 15o, (c) 30o, and (d) 45o at various times equals 5, 30, and 180 min with below cutoff wall opening S=4 cm
